# Enhancing alginate dialdehyde-gelatin (ADA-GEL) based hydrogels for biofabrication by addition of phytotherapeutics and mesoporous bioactive glass nanoparticles (MBGNs)

**DOI:** 10.1177/08853282241280768

**Published:** 2024-09-21

**Authors:** Faina Bider, Chiara Gunnella, Jana T Reh, Corina-Elena Clejanu, Sonja Kuth, Ana M Beltrán, Aldo R Boccaccini

**Affiliations:** 1 Institute of Biomaterials, 537499 Friedrich-Alexander University Erlangen-Nuremberg, Erlangen, Germany; 2 Department of Electronics, Information and Bioengineering, Politecnico di Milano, Milano, Italy; 3 Departamento de Ingeniería y Ciencia de los Materiales y del Transporte. Escuela Politécnica Superior, Virgen de África 7, Universidad de Sevilla, Seville (Spain)

**Keywords:** Hydrogels, bioactive glass particles, phytotherapeutic agent, 3D (bio)printing, drug delivery, bone tissue engineering

## Abstract

This study explores the 3D printing of alginate dialdehyde-gelatin (ADA-GEL) inks incorporating phytotherapeutic agents, such as ferulic acid (FA), and silicate mesoporous bioactive glass nanoparticles (MBGNs) at two different concentrations. 3D scaffolds with bioactive properties suitable for bone tissue engineering (TE) were obtained. The degradation and swelling behaviour of films and 3D printed scaffolds indicated an accelerated trend with increasing MBGN content, while FA appeared to stabilize the samples. Determination of the degree of crosslinking validated the increased stability of hydrogels due to the addition of FA and 0.1% (w/v) MBGNs. The incorporation of MBGNs not only improved the effective moduli and conferred bioactive properties through the formation of hydroxyapatite (HAp) on the surface of ADA-GEL-based samples but also enhanced VEGF-A expression of MC3T3-E1 cells. The beneficial impact of FA and low concentrations of MBGNs in ADA-GEL-based inks for 3D (bio)printing applications was corroborated through various printing experiments, resulting in higher printing resolution, as also confirmed by rheological measurements. Cytocompatibility investigations revealed enhanced MC3T3-E1 cell activity and viability. Furthermore, the presence of mineral phases, as confirmed by an in vitro biomineralization assay, and increased ALP activity after 21 days, attributed to the addition of FA and MBGNs, were demonstrated. Considering the acquired structural and biological properties, along with efficient drug delivery capability, enhanced biological activity, and improved 3D printability, the newly developed inks exhibit promising potential for biofabrication and bone TE.

## Introduction

The goal of tissue engineering (TE) is the regeneration of new tissues by the smart combination of cells, biomaterials and growth factors.^1^ Especially, the field of bone TE is currently in an exciting phase marked by significant research efforts directed towards the development of innovative and improved regenerative biomaterials.^2^ To address the demand for innovative medical technology and alternative approaches in TE, three-dimensional (3D) scaffolds continue to be at the centre of research. One method currently employed for producing 3D scaffolds is 3D bioprinting.^3^ This method involves an additive manufacturing process that requires the use of a 3D bioprinter and suitable biomaterials (bioinks) to construct 3D scaffolds through a layer-by-layer extrusion process.^4^ Bioinks typically consist of hydrogels derived from biocompatible polymers, chosen for their notable attributes such as appropriate mechanical properties, biodegradability, and printability. Additionally, bioinks must establish a suitable environment for cell proliferation and attachment, facilitating the development of 3D constructs that mimic human native tissue.^5^ Nevertheless, achieving an optimal balance between suitable printing characteristics, mechanical stability, degradability, and biocompatibility of bioinks is still challenging. Chitosan, collagen, gelatin and alginate are natural polymers frequently used to develop bioinks for 3D bioprinting. These materials exhibit noteworthy properties that make them suitable for replicating the characteristics of the native extracellular matrix (ECM).^6^ Alginate, a natural polymer extracted from brown algae, is employed in drug delivery strategies and 3D printing approaches.^7,8^ The alginate polymer chain consists of polysaccharide, forming a linear structure with (1,4)-linked β-D-mannuronic (M) and α-L-guluronic acid (G) residues. The G blocks have the potential to undergo ionic crosslinking in the presence of divalent cations such as strontium (Sr^2+^) or Ca^2+^, resulting in the formation of hydrogels.^7^ Furthermore, alginate is recognized for its widespread availability, excellent biocompatibility, and relatively low cost.^8,9^ However, the slow degradation rate of alginate hydrogels poses a limitation. This challenge has been addressed by utilizing the oxidized form of alginate known as alginate dialdehyde (ADA).^10^ As a result of the oxidation process, there is a reduction in molecular weight, leading to an accelerated degradation rate compared to unmodified alginate. Additionally, the oxidation process generates free aldehyde groups, allowing them to form bonds with amino (NH_2_)-groups in protein molecules, such as gelatin (GEL), through Schiff’s base formation.^8,11^ GEL is both bioresorbable and biocompatible, containing arginine–glycine**–**aspartic acid (RGD) sequences. The presence of RGD sequences in gelatin gives it the ability to enhance cell attachment and facilitate cell-cell interaction.^12^ Microbial transglutaminase (mTG) is an enzyme naturally present in the human body, playing a role in aiding blood coagulation and stabilizing protein structures.^13,14^ mTG assists in the crosslinking of structural proteins by creating bonds between the NH_2_-groups of glutamine and lysine in collagen, resulting in a suitable crosslinker for GEL.^13,15^

In recent times, there has been a growing interest in integrating herbal drugs into engineered biomaterials, due to the acknowledged biological functionalities of these phytotherapeutic agents.^16^ Specifically, the inclusion of compounds with phenolic rings, such as ferulic acid (FA), emerges as a favorable and promising strategy.^17^ FA (4-hydroxy-3-methoxycinnamic acid) is a naturally occurring compound found in plants, present in leaves, seeds, and plant cell walls. It has the capability to form covalent crosslinks with protein groups, such as lysine, that are present in GEL.^18,19^ Moreover, FA is widely recognized for its antiaging, anticancer, antidiabetic, antioxidative, and antibacterial characteristics.^17^ In our previous study^20^ the antibacterial effect of FA incorporated in ADA-GEL hydrogels was already proven using *S. epidermidis* strains. A reduction of colony forming units (CFU) on agar plates due to the addition of FA was found. A turbidity test with *S. aureus* as Gram-positive bacteria and *E. coli* as Gram-negative bacteria has shown the antibacterial effect of FA within ADA-GEL hydrogels. Moreover, FA has demonstrated potential in TE, especially for applications like skin regeneration or in skincare formulations. On the other hand, the exploration of multifunctional scaffolds incorporating FA in hydrogels, particularly for bone TE, has not been extensively investigated. To the best of our knowledge, only one publication by Anjali et al.^21^ has reported the integration of FA into a self-crosslinking ADA-GEL system, utilizing it as an antimicrobial compound for exudating wounds.^21^ In this work FA was incorporated into ADA-GEL-based hydrogels, emphasizing the significance of phytotherapeutic agents within hydrogels for biomedical applications and expanding the understanding of such composites. Furthermore, this work aimed to address specific challenges associated with natural hydrogels, such as mechanical weakness and limited bioactive properties. To overcome this limitations, we incorporated inorganic bioactive nanoparticles into ADA-GEL-based-hydrogels. These nanoparticles not only have the potential to enhance the mechanical properties of hydrogels but also may stimulate the nucleation and subsequent growth of apatite crystals on the surface of scaffolds, mimicking bone.^22–24^ A promising class of inorganic particles are mesoporous bioactive glass nanoparticles (MBGNs) in the SiO_2_-CaO system, which are produced using sol-gel approaches.^25,26^ Generally, bioactive nanoparticles find applications in hard TE, as carriers for drug delivery, or as coatings for implants, particularly due to their biocompatibility and osteostimulating properties.^25,27,28^ In this work, MBGNs (nominal composition: 70% SiO_2_, 30% CaO, mol%) were utilized, which are known for their significantly large pore volume and specific surface area. Additionally, these particles can be synthesized in customized sizes and shapes and they can be loaded with a suitable quantity of biomolecules or therapeutic drugs, facilitating their controlled release.^29,30^ The composition of SiO_2_-CaO nanoparticles is conducive to promoting osteoregeneration.^31^ In recent years, there has been significant work in the field of advanced bioinks for biofabrication, including the incorporation of phytotherapeutic agents and inorganic fillers into hydrogel bioinks. Several research groups have successfully demonstrated the efficacy of enhancing ADA-GEL hydrogels through the incorporation of bioactive glass nanoparticles. For example, Sarker et al.^32^ demonstrated that the incorporation of silicate bioactive glass into ADA-GEL may improve the mechanical strength of scaffolds.^32^ Leite et al.^24^ utilized 3D bioprinting to create ADA-GEL constructs reinforced through the incorporation of bioactive glass nanoparticles. These nanoparticles were found to induce the formation of an apatite layer, mimicking the native bone structure upon immersion into a simulated body fluid (SBF).^24^ Monavari et al.^33^ created 3D printed ADA-GEL scaffolds with bioactive glass particles loaded with icariin, which were able to increase the activity of osteoblast cells, resulting in improved cell proliferation, adhesion, and differentiation.^33^ In this study, our primary focus was on examining the improvements that should result from the simultaneous inclusion of FA and MBGNs (in two different concentrations) within ADA-GEL hydrogel. More precisely, we assessed the effect on degradation and swelling behaviour, as well as mechanical degradation through compression tests. Additionally, we investigated the release profiles of FA, Ca, and GEL from ADA-GEL-based samples. Since the intended application of the composite hydrogels is in the field of 3D (bio)printing, we performed degradation/swelling and release studies with 3D printed scaffolds (in addition to cast films). To assess the suitability of our inks for 3D (bio)printing, we conducted rheological measurements and 3D printing experiments on all inks. In addition to the filament fusion test (FFT), filament collapse test (FCT), and grid structure test (GST), we printed complex structures, including higher and larger scaffolds, bone-shaped and star-shaped designs, using the most promising inks. Furthermore, to explore the impact of FA and MBGNs on Schiff’s base formation in ADA-GEL, we performed a degree of crosslinking test. To demonstrate the possible mineralization resulting from the MBGN content, bioactivity tests were carried out by immersing samples in a SBF solution for 28 days. In vitro cytocompatibility studies were also conducted with the pre-osteoblastic cell line MC3T3-E1 on all inks. Additionally, we examined the release of vascular endothelial growth factor-A (VEGF-A), providing initial insights into the dual effect of FA and MBGNs on ADA-GEL bioinks properties.

## Materials & methods

### Materials

GEL from porcine skin (Type A, gel strength 300 bloom), commercially available high purity (pharmaceutical grade) alginate acid powder (sodium salt from marine brown algae, Vivapharm, Germany), calcium chloride dehydrate (CaCl_2_), sodium (meta)periodate (NaIO_4_), ethylene glycol and trans-FA (trans-4-Hydroxy-3-methoxycinnamic acid, 99%), sodium hydrogen carbonate (NaHCO_3_), and trinitrobenzenesulfonic acid (TNBS) were purchased from Sigma-Aldrich (Germany). Chemicals used for the sol-gel synthesis of MBGNs: cetyltrimethylammonium bromide (CTAB), ethyl acetate (EA), tetraethyl orthosilicate (TEOS, ≥99%), and calcium nitrate tetrahydrate (CN) (Ca(NO_3_)_2_·4H_2_O, ≥99%), were purchased from Sigma-Aldrich (Germany). Moreover, ammonium hydroxide solution (NH_4_OH, 28% basic solution) and ethanol (99.8%) were purchased from VWR International (Germany). Dulbecco´s Phosphate Buffered Saline (DPBS, [-] Ca^2+^, [-] Mg^2+^), Hank’s Balanced Salt Solution (HBSS); Calcein AM and 4′,6-Diamidin-2-phenylindol (DAPI), alpha-Modified Eagle’s medium (α-MEM) without ribonucleosides and depxyribonucleosides, penicillin-streptomycin (PS) and L-Glutamine, were obtained from ThermoFisher, Invitrogen (Germany). Microbial transglutaminase (mTG) was obtained from Ajinomoto Foods, Europe.

### ADA synthesis

The ADA used in this study was synthesized following a procedure described originally by Sarker et al.^34^ Briefly, 10 g of sodium alginate were suspended in 10 mL of ethanol. Following this, 1.337 g of NaIO_4_ were dissolved in 50 mL of MilliQ water, and the resulting solution was added to the alginate-ethanol suspension. This mixture was stirred for a duration of 6 h subsequently being quenched with 10 mL of ethylene glycol, followed by an additional 30 min of stirring. After 30 min, the stirring was stopped to facilitate the sedimentation of the ADA. The resultant material underwent a 3 day process of dialysis against MilliQ water and was subsequently freeze-dried.

### Synthesis of MBGNs

MBGNs with a nominal composition of 70% SiO_2_ and 30% CaO (mol%) were synthesized using a microemulsion based sol-gel method following the approach reported elsewhere.^35^ For this, 2.8 g CTAB was dissolved in 132 mL deionized water at 35°C. Subsequently, EA was added while lowering the temperature to 25°C for 30 min. After 30 min of stirring 28 mL of 1 M aqueous NH_4_OH was added and stirred for 15 min. Then, 14.4 mL TEOS was added and stirred for 30 min, followed by the addition of 9.12 g of CN. The solution was stirred for 4 h. After 4 h, the particles were collected using a centrifuge, washed 3 times with deionized water and once with ethanol (99.8%). The particles were left to dry overnight in an oven at 60°C, after which they were calcinated at 700°C for 5 h and 35 min with a heating rate of 2°C/min. The temperature was kept constant for another 3 h.

### Hydrogel preparation

**Figure 1.**
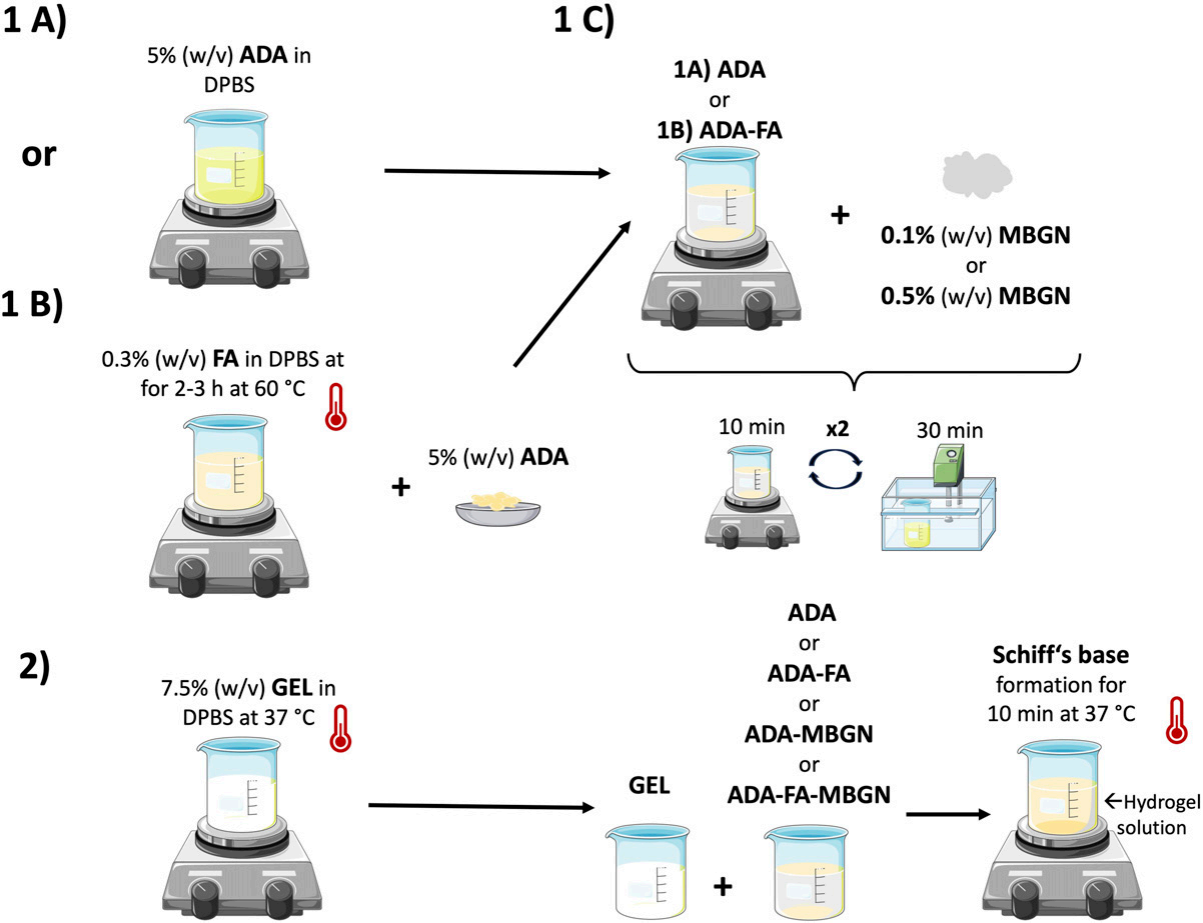
Schematic illustration of the preparation of ADA-GEL-based hydrogels.

To formulate ADA-GEL, 5% (w/v) lyophilized ADA was dissolved at RT and 7.5% (w/v) GEL at 37°C in DPBS. The solutions were mixed in 1:1 ratio, see Figure 1 (1A and 2) for 10 min to create 2.5%ADA-3.75%GEL. For ADA-GEL-FA samples the FA concentration was set to 0.15% (w/v) based on our previous results.^20^ Briefly, 0.3% (w/v) FA powder was dissolved in DPBS at 60°C for 2–3 h under continuous stirring until it was fully dissolved. Subsequently, ADA was added to the FA-DPBS solution and stirred until achieving a homogenous solution. Lastly, GEL was added to the ADA-FA solution (Figure 1 (1B and 2)) to create 2.5%ADA-3.75%GEL-0.15%FA hydrogel precursor. To prepare ADA-GEL-MBGN or ADA-GEL-FA-MBGN hydrogels, 0.1% (w/v) and 0.5% (w/v) of MBGNs were added to ADA or ADA-FA (see Figure 1 (1C)). To ensure the best distribution of particles, the mixture was stirred for 10 min and placed into an ultrasonic bath for 30 min. This step was repeated twice. When particles distribution was achieved, GEL was added to the ADA-MBGNs or ADA-FA-MBGNs mixture (see Figure 1 (1C and 2)). All used inks in this study are listed in Table 1.

**Table 1.** Composition and designation of all used inks.

ADA (w/v) (%)	GEL (w/v) (%)	FA (w/v)	BG (w/v)	Designature
2.5	3.75	-	-	ADA-GEL
2.5	3.75	0.15%		ADA-GEL-FA
2.5	3.75	-	0.1%	ADA-GEL-0.1%MBGN
2.5	3.75	-	0.5%	ADA-GEL-0.5%MBGN
2.5	3.75	0.15%	0.1%	ADA-GEL-FA-0.1%MBGN
2.5	3.75	0.15%	0.5%	ADA-GEL-FA-0.5%MBGN

### Fabrication of films

**Figure 2.**
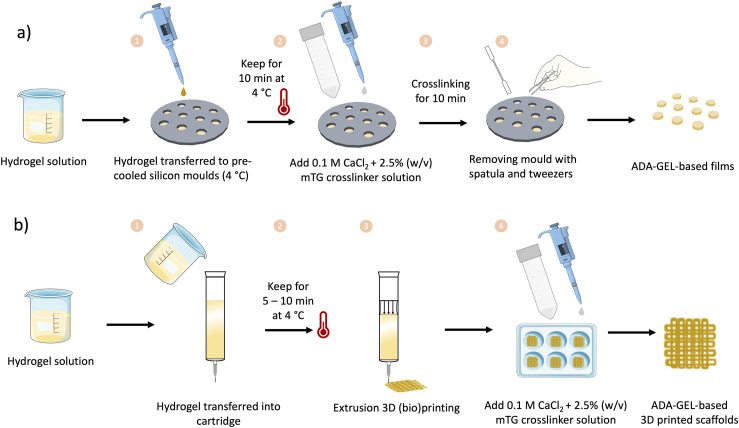
(a) Schematic illustration of the preparation of ADA-GEL-based films. (b) Schematic illustration of the preparation of ADA-GEL-based 3D printed scaffolds.

In Figure 2(a), a schematic illustration of the preparation of ADA-GEL-based films is shown. In order to prepare ADA-GEL films with/without FA and MBGNs, the hydrogel solution (preparation described in the previous section) was added into pre-cooled cylindrical silicon moulds with a diameter of 12 mm and a height of 2 mm. An exception in regard to size was made for the microtester compression measurements, where a silicon mould with a diameter of 5 mm and a height of 2 mm was used. To ensure the thermal gelation of GEL all moulds were placed into a 4°C fridge for 10 min. Subsequently, the hydrogel films were crosslinked with a crosslinking solution (2.5% (w/v) mTG in a 0.1 M CaCl_2_) for 10 min. During the crosslinking time, the films were detached from the silicon mould with a spatula. For sterile composite films preparation, MBGNs were heat sterilized for 2 h at 160°C prior to mixing and all hydrogels were sterilized by sterile filtrations with 0.22 µm (for GEL) and 0.45 µm (for ADA and ADA-FA) pore size membrane filters under sterile conditions.

### Fabrication of 3D constructs/3D printing

In Figure 2(b) the fabrication of 3D printed constructs with all hydrogels is displayed. Briefly, all inks were transferred into cartridges prior to printing, which were placed into a 4°C fridge for 5 – 10 min to achieve a suitable viscosity of GEL (due to the gelation process of GEL). The inks were extruded with a 3D printer (BioScaffolder 3.1, GeSim) layer by layer to create 4-layered grid-like structures using a nozzle with a diameter of 250 µm and a printing speed of 7 mm/s. All printing parameters were adjusted with the grid structure test (GST, described in detail further below) and kept constant throughout the sample preparation.

### Physicochemical characterization

Scanning electron microscopy (SEM, AURIGA®, Zeiss) images of MBGNs were obtained to characterize the morphology, distribution, and size of MBGNs. For SEM observation, particles were added homogenously on top of a carbon tape. To investigate the elemental composition of MBGNs energy dispersive spectroscopy (EDS, X-MaxN Oxford Instruments, UK) was performed using 10 kV voltage and a 6 mm working distance during SEM observations. Moreover, transmission electron microscopy (TEM, TALOS F200S, Thermo Fischer Scientific, USA) was used to observe the microstructure of MBGNs. Moreover, the MBGNs were characterised using an ART-FTIR spectroscopy (IRAffinity-1S, Shimadzu, Germany) in order to investigate the surface chemistry. The measurements were performed in absorbance mode from wavenumber 4000 to 400 cm^−1^ by applying 40 scans with a resolution of 4 cm^−1^.

### *In vitro* degradation/swelling behaviour

A degradation/swelling study was conducted to examine the influence of FA and MBGNs on such behaviour. ADA-GEL films and/or 3D printed scaffolds (*N* = 6, respectively) of all compositions (listed in Table 1) were assessed for their weight changes over a 28 day incubation period in cell culture medium. Briefly, sterile conditions were maintained while preparing films with dimension of 12 mm wide and 2 mm height and/or 1 × 1 cm^2^ 3D printed scaffolds for each composition. All samples were placed into pre-weighed inserts, which were then positioned in a 6-well plate and covered with cell culture medium. The samples were stored under cell culture conditions at 37°C and 5% CO_2_ with the medium refreshed twice a week. Weight measurements were taken within the first 3 h at 15-min intervals, and subsequently after 24 h, 3, 7, 14, 21, and 28 days. The percentage change in weight (W_r_) was calculated using the following equation:

(1) $$W_{r}=\left(\frac{W_{t-}W_{0}}{W_{0}}\right)\times 100$$

where W_0_ represents the initial weight of the samples before immersion in the cell culture medium, and W_t_ denotes the current weight of the samples after immersion into the cell culture medium.

### Drug release studies

#### FA release

The impact of the MBGN content on FA release from ADA-GEL-based films and/or scaffolds was determined by a FA release study. Briefly, 6 sterile ADA-GEL-FA, ADA-GEL-FA-0.1%MBGN and ADA-GEL-FA-0.5%MBGN films and 1 × 1 cm^2^ 3D printed scaffolds were prepared and printed, respectively, located into 6-well plates and covered with HBSS. ADA-GEL-FA samples were used as reference material. The FA release was measured cumulative via UV-Vis spectrometer (Specord40, Analytic Jena, Germany) at a wavelength of 310 nm and using the software WinASPECT 2.5.8.0. The absorbances were measured after 24 h, 3, 7, 14, 21 and 28 days of incubation. Therefore, at each time point 1 mL of HBSS supernatant was collected for the measurement and replaced with fresh HBSS. The FA release was measured in µg/µl.

#### Ca release

The calcium release out of films and 3D printed scaffolds was measured with a calcium colorimetric assay kit (MAK022, Sigma-Aldrich, Germany). With this kit, the calcium ion concentration can be measured based on chromogenic complexes of calcium ions attaching to *o*-cresolphthalein, which is proportional to the concentration of calcium ion. Briefly, 90 µl of chromogenic reagent and 60 µl calcium assay buffer were added to a 96-well plate containing 50 µl of sample. The experiment was carried out at RT and under the absence of light, due to the light sensitivity of the assay. After 5 – 10 min incubation at RT a colour change to violet occurred thereafter the absorbance was measured at a wavelength of 575 nm via a microplate reader (PHOmo reader, Autobio Diagnostics Co., Ltd). The concentration of calcium was calculated using the following equation:

(2) $$Concentration\;of\;calcium=\frac{S_{a}}{S_{v}}$$

where S_a_ is the unknown calcium amount [µg] taken out of the prior prepared standard curve and S_v_ is the volume of the samples, which is in this case 50 µl.

#### GEL release

To investigate the GEL release out of films and 3D printed scaffolds a colorimetric protein assay, such as the Coomassie blue G-250 dye-binding (Bradford) assay was carried out. The Bradford protein assay is a frequently used colorimetric method to detect the protein (GEL) concentration. Therefore, a calibration curve was prepared prior the experiment with known GEL concentrations. By mixing the samples with Bradford solution a colour reaction change to blue occurs. The intensity of the colour change is proportional to the content of aromatic and basic amino acids. The optical absorbance was measured by UV-Vis spectroscopy at a wavelength 595 nm.

### Degree of crosslinking

In order to investigate the impact of MBGNs on the Schiff’s base formation of ADA-GEL-based hydrogels, a trinitrobenzenesulfonic acid (TNBS) assay was performed. This experiment allows the determination of free amino acids (NH_2_-groups) of GEL. If ADA (free aldehyde groups) is present, a reversible Schiff’s base formation occurs which reduce the presence of NH_2_-groups in ADA-GEL samples. As reference materials 3.75% (w/v) GEL samples (positive control) and pure ADA 2.5% (w/v) samples (negative control) were used. The assay was conducted following previous work reported by Nguyen et al.^36^ Briefly, ADA, GEL, ADA-GEL, ADA-GEL-0.1%MBGN, ADA-GEL-0.5%MBGN, ADA-GEL-FA-0.1%MBGN and ADA-GEL-FA-0.5%MBGN hydrogels were prepared, frozen and freeze dried (*N* = 5 respectively). Subsequently, 5 mg of the lyophilized material was solved in 1 mL of 4% solution of NaHCO_3_ at 60°C with the help of a vortex mixer. Next, 1 mL of a 0.5% (v/v) solution of yellow TNBS was added to each sample and vortexed again. A blank sample was used as a reference during the UV-Vis measurements. Subsequently, samples were stored in a shaking incubator for 4 h and 60°C. Within the 4 h period, a colour change from yellow to orange occurred. After incubation, 1 mL of each solution was added to 3 mL of 6 M HCL solution and incubated in the shaking incubator for further 1.5 h at 40°C. During the 1.5 h period, the colour changed from orange to yellow. Lastly, 1 mL of each sample was transferred into UV-cuvettes, whereas the blank samples were set as the reference material. The measured absorbances were detected by a UV-Vis spectrometer at 346 nm. It is known that the higher the absorbances the higher is the amount of free NH_2_-groups and the lower is the degree of crosslinking (%D_c_). The %D_c_ was recalculated using the following equation:

(3) $${\%D}_{c}=\frac{A_{uncrosslinked}-A_{crosslinked}}{A_{uncrosslinked}}\times 100$$

### HAp formation

The HAp formation ability on the surface of ADA-GEL samples containing MBGNs was assessed by conducting a bioactivity study over 28 days of immersion in a simulated body fluid (SBF) solution.^37^ Films were immersed within a certain amount of SBF solution and incubated at 37°C with continuous shaking (80 rpm). The SBF solution was changed twice a week to simulate conditions within the human body and to maintain the ionic concentration within the SBF solution. Four replicates were prepared for each time point. ADA-GEL-0.1%MBGN, ADA-GEL-0.5%MBGN, ADA-GEL-FA-0.1%MBGN and ADA-GEL-FA-0.5%MBGN hydrogels were used to fabricate films of dimensions 12 mm × 2 mm. Films were taken out of the SBF solution (25 mL) after 1, 3, 7, 14, 21 and 28 days and rinsed with distilled water. Two of these four ADA-GEL-based replicates with/without FA and with/without MBGNs were prepared for SEM using a SEM-FIX I and SEM-FIX II solution, composed of 0.1% glutaraldehyde and 2% paraformaldehyde and 0.3% glutaraldehyde and 3% paraformaldehyde, respectively, each for 1 h. Afterwards, samples were immersed in an ethanol series (from 30% up to 99%) for 30 min each. Subsequently, the samples were dried using a critical point dryer (EM CPF3000, Leica) and fixed onto a carbon tape for SEM observation. The other two of the four replicates were freeze dried (freeze dryer, ALPHA 1-2 LDplus, CHRIST, Germany). In order to investigate the HAp formation on the surface of samples the freeze dried samples were analysed using ATR-FTIR spectroscopy in absorbance mode from wavenumber 4000 to 400 cm^−1^ with a resolution of 4 cm^−1^. XRD (MiniFlex 600, Rigaku) was also carried out to track possible changes in the crystalline phases. The XRD analysis was measured between 2θ 20° and 80° with a resolution of 0.02° and a scanning rate of 4°/min. Moreover, SEM observations and EDS analysis were performed in order to assess the surface morphology.

### Determination of ion release from MBGNs in SBF

The release of Si and Ca ions was measured by inductively coupled plasma-optical emission spectrometry (ICP-OES, Agilent 5100 SVDV). Briefly, MBGNs in concentration of 0.1% (w/v) and 0.5% (w/v) were immersed into 30 mL of SBF solution in falcon tubes. Tubes were incubated at 37°C in a shaking incubator (KS 4000 i control, IKA, Germany) at 80 rpm. After 7, 14 and 21 days of incubation in SBF, falcon tubes were centrifuged at 7830 rpm for 15 min and supernatants were collected. Supernatants were stabilized with concentrated HNO_3_ until a pH of ≤2 was reached and filtered using a 0.22 µm filter. Pure SBF solution without any MBGNs was used as reference solution. Prior to the analysis, three calibration solutions were formulated to establish a linear relationship between intensity and concentration. These calibration solutions were created using reference standards that are certified for ICP techniques. The time depended concentration release of Ca and Si ions into SBF medium was measured in cumulative form.

### Mechanical characterization

#### Compression test

To determine the effective modulus of films and to investigate the impact of MBGNs on mechanical properties, a test was performed in compression using a Microtester (Microtester LT, Cellscale). Samples (*N* = 4 per composition, compare Table 1) with a diameter of 5 mm and height of 2 mm were prepared in sterile conditions, placed into a 24-well plate, covered with cell culture medium and stored at 37°C and 5% CO_2_ humidity. The medium was changed 2 times a week. The measurements were performed after 24 h, 3, 7, 14, 21 and 28 days. Day 0 samples were measured directly after crosslinking and without any immersion in cell culture medium. The crosslinking solution was composed of 2.5% (w/v) mTG in 0.1 M CaCl_2_ solution. For the measurement a 6 mm × 6 mm plate and a cantilever beam with a diameter of 0.5588 mm were used. The test was set to 20% of displacement with 4 cycles per measurement. Each cycle consisted of 20 s loading time, 2 s of holding time and a recovery of 20 s with a final holding time of 2 s. The effective modulus for each sample was calculated by the slope of the 5% – 10% section of each obtained stress-strain curve. The force and maximum compression stress values were extracted from the obtained data.^38,39^

#### Rheological evaluation

Rheological measurements were used to investigate the impact of MBGNs on the viscosity of ADA-GEL-based hydrogels and therefore to assess the shape fidelity of the 3D printed scaffolds during the 3D (bio)printing process. All inks mentioned in Table 1 were used for the viscosity evaluation. For the measurements a discovery HR 3 rheometer (Discovery Hybrid Rheometers, TA instruments, USA) was used. This device is equipped with a cone plate geometry (cone of 2°). Prior to the measurements, an amplitude sweep was performed with pure ADA-GEL as a reference to adjust the linear viscoelastic region. Subsequently, frequency sweep tests were performed for all inks. All compositions were measured 3 times at RT. Briefly, 100 µl of each hydrogel was added to the lower plate of the rheometer. The amplitude sweep was performed with a constant frequency of 10 rad/s with a strain range of 1 to 200%. The frequency sweep was conducted in a frequency range of 0.1 to 100 Hz and at a constant elongation oscillation strain of 10%, which was prior determined.

### Assessment of 3D printability

**Figure 3.**
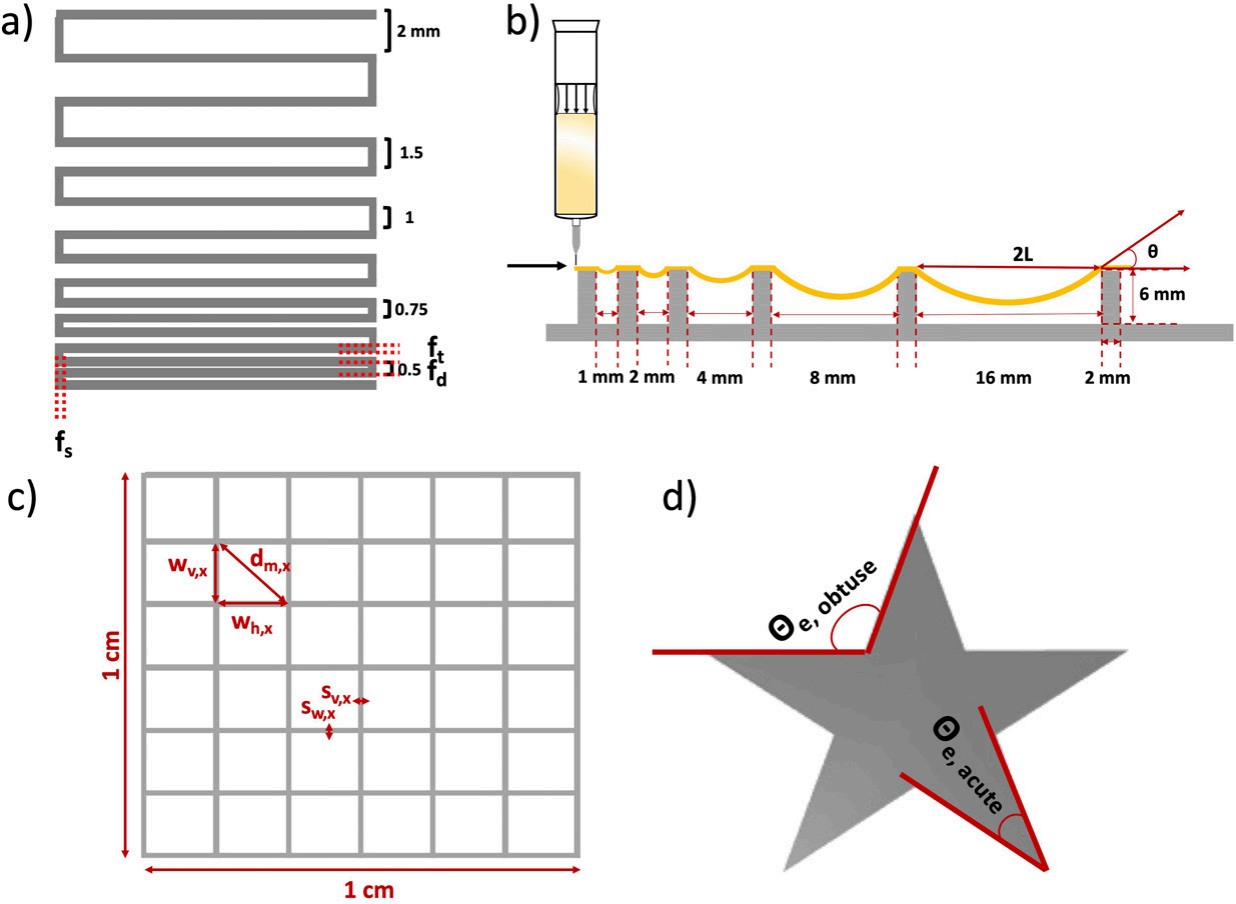
Schematic illustration of 3D printing experiments. (a) Illustration of FFT pattern. (b) The platform employed for the FCT featuring pillars with progressively increasing distances and showcasing the deflection angle of a printed filament navigating two obstacles. (c) GST, x was set to 5 measurements. (d) Complex star-shape printing and experimental angles for calculating the angular deviation.

The capability of hydrogels to be 3D printed was examined through various experiments conducted with a 3D printer (BioScaffolder 3.1, GeSim). All inks (see Table 1) were prepared as mentioned above. ImageJ software was used to evaluate the results of all 3D printing experiments. Figure 3 illustrates the performed 3D printing experiments, which are explained in detail below.

#### Filament fusion test (FFT)

The FFT was conducted to determine the achievable resolution of different inks. This parameter can be assessed as the minimum distance at which two adjacent strands merge. The test was inspired by the work of Ribeiro et al.^40^ The experiment involved printing a one-layered meandering pattern with gradually reducing distance between the strands, demonstrating the point at which two adjacent strands become indistinguishable. In Figure 3(a) a printed structure is depicted with a gradually changing distance between the strands, varying from 2, 1.5, 1, 0.75 and to 0.5 mm. The ratio between the fused segment length (f_s_) and the filament thickness (f_t_) was evaluated and plotted against the filament distance (f_d_), as commonly reported in the literature.^41^

#### Filament collapse test (FCT)

The FCT proves valuable in determining the maximum distance between two objects where a printed strand remains intact without collapsing. This experiment is based on the idea introduced by Ribeiro et al.^40^ and Therriault et al.^42^ This test is particularly crucial for porous structures, ensuring that strands over pores do not tear down. To conduct the test, the ink is printed over a structure featuring pillars positioned at increasing distances, as illustrated in Figure 3(b). The deflection angle was assessed for three samples of each composition using ImageJ software.

#### Grid structure test (GST)

The idea for the GST was acquired from previous work reported by Hazur et al.^43^ Briefly, a 1 × 1 cm^2^ grid (see Figure 3(c)) composed of four layers was printed. The pressure was kept constant in the range of 15 ± 10 kPa in order to compare the geometry of strands and pores of all printed compositions. All 3D printed scaffolds were imaged with a dark field microscope (Stemi 508, Carl Zeiss, Germany). The microscopy images were evaluated using ImageJ, where calculations were performed to determine the strand width vertically (s_v_) and horizontally (s_h_), as well as the pore width vertically (w_v_) and horizontally (w_h_) (marked in Figure 3(c)). Each 3D printed scaffold was measured five times within the same image. The ideal diagonal of a pore was then established by the following equation:

(4) $$d_{ideal}=\sqrt{w_{v}^{2}+w_{h}^{2}}$$

This value was then compared with the actual diagonal (d_m_) using the diagonal correlation rate (DCR):

(5) $$DCR=\frac{d_{m}}{d_{ideal}}$$

#### Complex star-shape printing

Complex structure printing was performed for ADA-GEL-FA and ADA-GEL-FA-0.1%MBGN, which were the most promising (bio)inks (see the Results section below). In particular, star-shaped structures were printed as described by Wu et al.^44^ The quantitative test involves the evaluation of the angular deviation (D_a_) between the theoretical angle (θ_t_) (acute and obtuse) and the experimental angle (θ_e_) (acute and obtuse) measured using ImageJ:

(6) $$D_{a}=\frac{\theta_{e}-\theta_{t}}{\theta_{t}}$$

The experimental angle (θ_e_) was calculated for both acute and obtuse angles by drawing a line connecting the vertex to the intersection (see Figure 3(d)). The theoretical angle (θ_t_) was set at 36° for the acute angle and 108° for the obtuse angle.^44^

### *In vitro* cytocompatibility

In order to analyse the impact of MBGNs and FA on cell viability, a cytotoxicity test was performed. For this purpose, the undifferentiated preosteoblastic cell line MC3T3-E1 was used. Pure ADA-GEL hydrogel was used as a reference material. For all hydrogel compositions (see Table 1) 1 × 10^6^ cells/ml were incorporated. After the sample preparation (films and/or 3D bioprinted scaffolds), all samples were crosslinked with a crosslinking solution composed of 2.5% (w/v) mTG in 0.1 M CaCl_2_ solution for 10 min. Thereafter, all samples were covered with α-MEM cell culture medium (details mentioned below) and stored in an incubator under cell conditions.

#### Direct cell culture test

In order to perform direct cell culture tests, 50 µl of each MC3T3-E1 cell laden hydrogel was pipetted into each well of a 48-well plate, crosslinked, washed and covered with cell culture medium until further stainings (Calcein AM and DAPI). Stainings were performed after 24 h and 7 days of incubation. Cell culture medium was changed twice a week. Pure ADA-GEL samples without any cells were prepared as reference.

#### 3D bioprinting

For the 3D bioprinting process, all cell laden ADA-GEL hydrogels (listed in Table 1) were transferred into cartridges and installed into the GeSim 3D printer described above. Subsequently, 4 layers scaffolds with a size of 1 × 1 cm^2^ were printed into 6-well plates (*N* = 6).

#### Cell culture

For the preosteoblastic cell line MC3T3-E1 a α-MEM medium with 1.0% (v/v) PS, 1.0% (v/v) L-Glutamine and with 10% (v/v) of Fetal Bovine Serum (FBS, Sigma-Aldrich, Taufkirchen, Germany) was needed. Cells were incubated at 37°C, 5% CO_2_ and 95% relative humidity, and were splitted/used for experiments after reaching a confluency of 80% – 90%.

#### Cell viability

The viability of MC3T3-E1 cells embedded in all tested inks was determined by using a WST-8 cell counting kit (Cell-Couning-Kit8, Sigma-Aldrich, Germany). Thereby, a 5% (v/v) WST-8 solution was used. The absorbances were measured after 4 h of incubation with a plate reader (PHOmo microplate reader, China) at a wavelength at 450 nm. The activity of cells correlates with the intensity of the measured absorbance through a calibration curve.

#### Live/dead staining

Subsequent to the WST-8 test, a live/dead staining was carried out. For the live staining Calcein AM (Invitrogen, Germany) was used, whereas for dead cell staining DAPI (Invitrogen, Germany) was used. Calcein AM is a staining which is used to investigate the amount of living cells and DAPI is used to assess the nuclei of cells resulting in the total amount of cells. More detailed, after discarding the WST-8 solution and a subsequent washing step, the Calcein AM solution in a 4 µl/ml concentration in HBSS was added to the samples for 45 min in dark conditions. After 45 min incubation time, the Calcein AM solution was discarded, washed and fixed with a 3.7% formaldehyde solution in HBSS for 10 min. After the fixation, a 1 µl/ml DAPI solution in HBSS was added to the samples for 15 min. After 15 min, DAPI solution was discarded, samples were washed and slightly covered with HBSS for further fluorescence microscopy investigation. For imaging, a fluorescence microscope (Axio Scope A1, Carl Zeiss, Germany) was used. Each composition was prepared in *N* = 3 samples/wells. Each well was imaged 3 times resulting in 9 images per sample. The viability of cells was calculated according to the following equation:

(7) $$Cell\;viability\;\left[\%\right]=\left(\frac{Surviving\;cells}{Total\;amount\;of\;cells}\right)\times 100$$

For the counting of alive cells (green) and for the total number of cells (blue) the software ImageJ and the associated plugin “ITCN” were used.

### Vascular endothelial growth factor-A (VEGF-A) release

The angiogenic capability of ADA-GEL-based hydrogels with FA and MBGNs was assessed using a mouse VEGF Elisa Kit (RayBiotech, Inc., USA) and by measuring the secretion of crucial biological markers such as the vascular endothelial growth factor (VEGF-A) by MC3T3-E1 cells. For this test, 0.5 mL ADA-GEL hydrogels with/without FA and MBGNs (*N* = 3) were transferred into 24-well plates. Afterwards, the samples were crosslinked with a crosslinking solution composed of 0.1 M CaCl_2_ solution with 2.5% (w/v) mTG. Subsequently, each sample was seeded with 15,000 MC3T3-E1 cells and with associated cell culture medium for 1 day under cell culture conditions at 37°C. Pure cells were seeded on top of the plastic well (without hydrogel) as reference material (control). After 24 h of incubation time, the cell culture medium was replaced with fresh (1 mL) cell medium and further incubated for 72 h. After 72 h the medium was collected and stored in the freezer until further use. The viability of cells was confirmed by a subsequent WST-8 analysis on top of each sample. For the VEGF-A experiment, 100 µl of each supernatant solution was used following the ELISA protocol. Finally, the optical density was measured by a plate reader at 450 nm to quantify the VEGF-A release. The MC3T3-E1 VEGF-A release was calculated against a standard curve.

### ALP and Bradford analyses

The ALP and Bradford analyses were conducted to evaluate the osteogenic behaviour of ADA-GEL-based samples with/without FA and MBGNs. The ALP activity was measured photometrically after 7, 14 and 21 days after being cultivated in cell culture medium. Briefly, 0.5 mL of all inks (Table 1, *N* = 6 replicates) were filled in a 24-well plate with *N* = 6 for each time point and crosslinked with the crosslinking solution. After sample preparation, 15,000 MC3T3-E1 cells were seeded on top of each well/sample, covered with cell culture medium, and incubated under cell culture conditions. The medium was changed after 72 h to let the cells reach sufficient confluency on top of each sample. Subsequently, the medium was changed twice a week. At the mentioned time points, the medium was discarded, and cells were washed with HBSS. Afterwards, MC3T3-E1 cells were covered with 1 mL of a lysis buffer solution for 45 min, respectively. Lysis buffer was prepared using 2.5 mL of Triton X-100 with Tris (CarlRoth, Germany), MgCl_2_ and ZnCl_2_, which was filled up with ultrapure MilliQ water and stirred at RT. After 45 min the lysate solutions were collected in tubes and stored at −21°C until all samples were accumulated. After all samples for day 7, 14 and 21 were collected, they were taken out of the freezer to warm up to RT. All tubes were centrifuged for 5 min at 2000 rpm. 250 µl were mixed with 100 µl ALP mix solution, composed of ALP buffer (0.1 M Tris and 2 mM MgCl_2_ in ultrapure water) and 9 mM para-nitrophenyl phosphate (p-NPP, ThermoFisher, Germany) directly in a cuvette and incubated in dark condition at RT until a colour change to yellow occurred. The ALP activity was measured quantitively by absorbance intensity changes of p-nitrophenol which is dissociated by ALP enzymatically. After the colour change, the reaction was stopped by adding 650 µl NaOH (CarlRoth) into each cuvette. The time of colour change was noted. As a reference, 250 µl of ultrapure water was mixed with 100 µl ALP mix and 650 µl NaOH was used. Thereafter, optical absorbance was measured using UV-Vis spectroscopy (Analytik Jena AG, Germany) at a wavelength λ = 405 and 690 nm. The ALP activity is represented by the conversion of nanomolar p-NPP transformed to para-nitrophenol (n-NP) under the influence of the ALP secreted by the cells over time (per minute). This ALP activity was later normalized to the total protein content in order to measure the specific ALP activity. To determine the cells total protein content a Bradford analysis was carried out by mixing 25 µl of the lysate solution with 975 µl of Bradford reagent solution (AppliChem GmbH, Germany) within a cuvette. As reference, ultrapure MilliQ water was used instead of the lysate solution. The absorbance was measured with the UV-Vis spectrometer at λ = 595. A colour change from brown to blue could be observed.

### *In vitro* biomineralization

To visualize the deposition of HAp an Osteoimage fluorescent staining kit (Lonza, Germany) was used. For the experiment, 0.5 mL of all inks (Table 1, *N* = 3 replicates for each time point) were transferred into 24-well plates and crosslinked with the crosslinking solution as mentioned above. After sample preparation, samples were seeded with 15,000 MC3T3-E1 cells per well and covered with osteogenic medium composed of α-MEM cell culture medium and additional 10 mmol/l β-glycerophosphate, 0.05 mmol/l ascorbic acid, and 10 nmol/l dexamethasone (all purchased from Sigma-Aldrich). Afterwards, the osteogenic medium was changed twice a week. Wells with pure cells were used as a reference. The fluorescent specific experiment was performed after 7 and 14 days of incubation following the LONZA protocol. For each experimental day separate samples were prepared. After certain incubation time points, the medium was removed and washed with HBSS. Subsequently, the samples were fixed with 3.7% (v/v) paraformaldehyde solution containing a fixation buffer for 20 min. After the fixation, the samples were washed twice using the diluted washing buffer (1:10) provided by the manufacturer (LONZA). Subsequently, the samples were stained with the diluted staining solution (1:100) and incubated for 45 min in dark conditions. After 45 min, the stained reagent was discarded, and the samples were again washed with the diluted wash buffer solution. Finally, the samples were covered with the washing buffer solution and quantitatively assessed by a plate reader at 485/520 nm and observed by a florescence microscope.

### Statistical analysis

Statistical analysis was conducted using OriginLab software (OriginLab, 2021, USA) via a one-way ANOVA test along with the Bonferroni test for significance levels of *p* < 0.05 = *, *p* < 0.01 = ** and *p* < 0.001 = ***. N = number of technical replicates. *N* = 6 samples were used for release studies (FA, Ca and GEL) and degradation/swelling studies, *N* = 5 for the determination of degree of crosslinking, *N* = 4 replicates were set for compression testing and for the apatite formation study, *N* = 3 for the rheological measurements, grid structure test analysis, VEGF-A expression measurement, ALP/Bradford experiments and for the in vitro biomineralization experiments.

## Results and discussion

The main goal of this study was to improve the 3D printability of ADA-GEL hydrogels by the incorporation of FA and MBGNs. For all experiments, hydrogel films were prepared from all compositions mentioned in Table 1. To address the main application, 3D printed scaffolds with the most promising inks, namely, ADA-GEL, ADA-GEL-FA, ADA-GEL-FA-0.1%MBGN and ADA-GEL-FA-0.5%MBGN were additionally prepared and investigated in terms of a degradation study and the assessment of Ca, GEL and FA-release. For all experiments, ADA-GEL cast films and/or 3D printed ADA-GEL scaffolds were used as references.

### Physicochemical characterization

**Figure 4.**
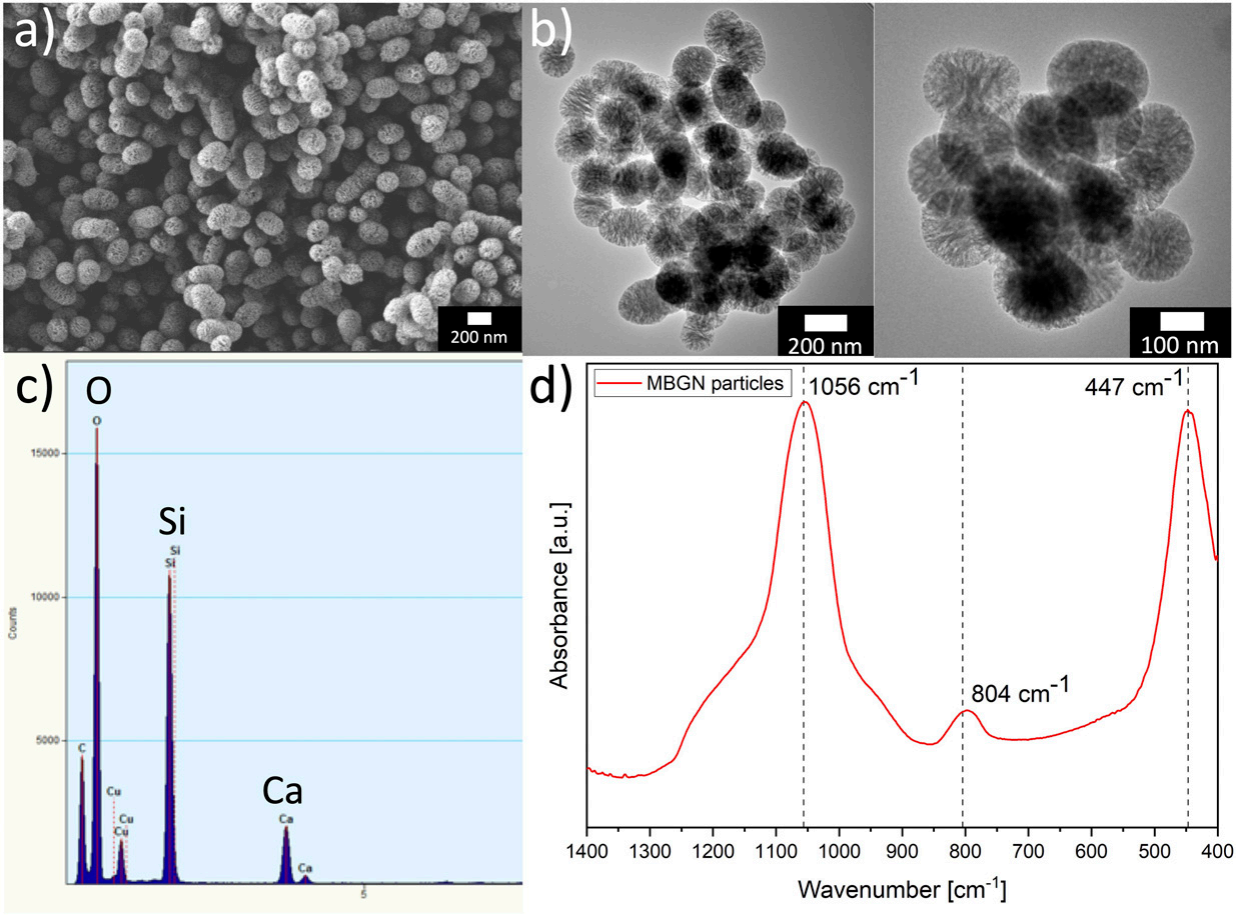
(a) SEM image (scale bar: 200 nm) and (b) TEM images (scale bars: 200 nm and 100 nm) showing the morphology and size distribution of MBGNs. (c) EDS (TEM) analysis confirming the presence of calcium and silicon. (d) ATR-FTIR spectra of MBGNs.

Figure 4(a) shows a SEM image and Figure 4(b) shows TEM images of the MBGNs used in this study. A particle size of ∼100 – 200 nm and homogeneous particle distribution are visible. The synthesis process also yielded a small number of ellipsoidal particles, which is attributed to the strong adherence of microemulsion templating droplets during manufacturing.^45^ Importantly, the observed particle size closely aligns with the dimensions previously reported for MBGNs synthesized using microemulsion-based techniques, reinforcing the consistency of this approach with established research findings.^45–47^ In Figure 4(c) EDS (TEM) peaks confirm the presence of Ca and Si in MBGNs with nominal composition of 70SiO_2_-30%CaO (mol%). Moreover, in Figure 4(d) typical ART-FTIR spectra of MBGNs are shown, corresponding to the distinctive Si-O-Si groups, indicating a rocking mode at 447 cm^−1^, a bending mode at 804 cm^−1^ and a stretching mode at 1056 cm^−1^, respectively.^48^

### *In-vitro* degradation/swelling behaviour

**Figure 5.**
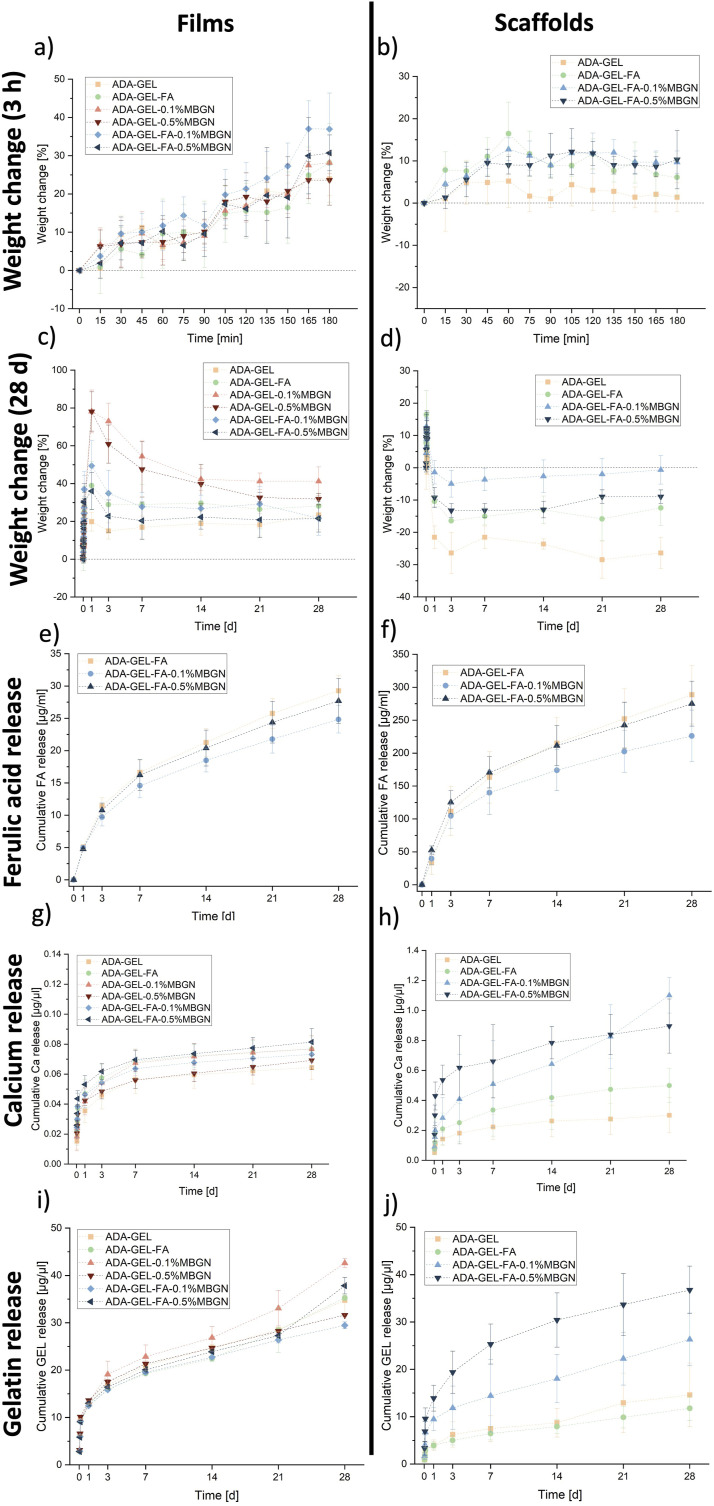
Degradation/swelling behaviour of films of all used hydrogel compositions and 3D printed ADA-GEL scaffolds with/without FA and MBGNs in (a) – (b) for the first 3 h and (c) – (d) after 28 days. Cumulative FA release from (e) films with/without MBGNs and (f) 3D printed ADA-GEL scaffolds with/without MBGNs over 28 days of incubation in HBSS measured at wavelength of 310 nm. Ca release out of (g) films of all used hydrogel compositions and (h) 3D printed ADA-GEL scaffolds with/without FA and MBGNs in [µg/µl]. Cumulative GEL release out of (i) films j) 3D printed ADA-GEL scaffolds with/without FA and MBGNs in [µg/µl].

The degradation/swelling behaviour of ADA-GEL-based films (compositions in Table 1) and 3D printed scaffolds with the most promising inks within the first 3 h is shown in Figure 5(a) and (b) and after 28 days of incubation in Figure 5(c) and (d). The control of the degradation behaviour is crucial for the application of the scaffolds in TE. On one hand, a too fast degradation of the scaffold material does not lead to an stabilizing structure for injured tissues and would result in a lack of ECM like structure surrounding cells during regeneration.^49^ Moreover, there can be the risk that the supportive structure might degrade faster than the regeneration of the native tissue, resulting in a reduced impact of the signal molecules, which were incorporated within the scaffold.^50^ On the other hand, a too slow degradation of the material would result in the inhibition of regeneration, as the needed space for new tissue formation would still be occupied by the biomaterial.^50,51^ Moreover, the degradation of the hydrogel has a significant influence on the release of FA, GEL and Ca (from MBGNs) (release studies are discussed further below). For that reason, the degradation/swelling study was performed. Additionally, an adapted degradation rate of hydrogels films and 3D printed scaffolds enable a suitable mesh size which leads to a successful cell penetration and attachment, as well as to an absorption of biological fluids required for nutrients exchange for cells.^52,53^ Besides, a control of material degradation can lead to a more predicable estimation of mechanical degradation (stiffness decrease), which is crucial for cell differentiation within the material.^54^ Among all compositions no significant differences was shown in regard to the weight change during the first 3 h of incubation of films (see Figure 5(a)). However, ADA-GEL-MBGN films’ weight increased more after 24 h of incubation (see Figure 5(c)). The presence of MBGNs presents an hydrophilic surface which is able to attract water molecules resulting in higher water uptake in comparison to pure ADA-GEL scaffolds,^24^ explaining the behaviour of ADA-GEL-MBGN films within the first 24 h. Interestingly, one can observe that ADA-GEL-FA-MBGN samples seem to change the weight in a more constant manner up to 28 days compared to pure ADA-GEL-MBGN films, which might be attributed to the additional crosslinking between FA and GEL^21^ resulting in a more dense structure and lower retention of water. For scaffolds, similar behaviour occurs during the first 3 h, except for the fact that the reference material (pure ADA-GEL) seems to have the lowest swelling ability. This behaviour can be related to the presence of FA which leads to additional crosslinking to GEL.^21^ The weight change for films reached values up to 78% after 24 h of incubation, whereas scaffolds to a max. of 15%. The difference in water uptake of scaffolds compared to films is understandable due to the reduced amount of material present in the 3D printed scaffold and therefore bigger surface to volume ratio compared to films resulting in less capability of maintain medium in the hydrogel. Moreover, the presence of open pore structure of the scaffolds is also leading to a faster weight loss. After reaching the highest weight change after 24 h, ADA-GEL-0.1%MBGN and ADA-GEL-0.5%MBN samples exhibited the highest weight loss from 1 day up to 7 days of incubation. From day 7, the samples appeared to be more stable in comparison to other compositions. The stability of samples after 7 days of incubation can be explained by the additionally crosslinking of the material induced by the released Ca ions from the MBGNs. Samples reached a weight change of 41% and 32%, respectively, similar to results shown elsewhere.^55^ Anions of the dissolved MBGNs can assist the interaction with peptide bonds and crosslinks of GEL, which is known as general-base catalysis resulting in stable ADA-GEL-MBGN samples.^56^ ADA-GEL-FA films showed a slower degradation behaviour compared to pure ADA-GEL films, which can be attributed to the additional crosslinking between FA and GEL.^21^ In our previous work on incorporation of FA into ADA-GEL hydrogels, an accelerated degradation behaviour of ADA-GEL-FA samples compared to neat ADA-GEL samples was shown.^20^ We explained this behaviour with a possible decrease of pH within the hydrogel due to the presence of FA. Nevertheless, with an improved handling of the sample preparation, a slower degradation behaviour of ADA-GEL-FA samples can be achieved. The degradation of ADA-GEL-based hydrogel films with MBGNs in general might be advantageous because of the release of ions which might lead to a higher osteoconductivity and osteogenic differentiation.^57^ The impact of the degradation behaviour of ADA-GEL-based hydrogels containing MBGNs, especially in terms of the formation of a HAp layer on the surface of the films (bioactivity), is discussed in more detail further below. The expected accelerated degradation of 3D printed scaffolds compared to films was confirmed in this study and might be explained by considering that the exposed surface of 3D printed grids is significantly higher than the ones of the films. The scaffolds are therefore more influenced by the external medium leading to a faster degradation kinetic.^58^ Moreover, for both films and scaffolds, with increasing MBGN concentration a faster degradation was determined. ADA-GEL-0.1%MBGN and ADA-GEL-FA-0.1%MBGN films and scaffolds seemed more stable during the incubation time compared to samples with 0.5% (w/v) MBGNs. A possible explanation for this behaviour might be the fact that a high concentration of MBGNs likely hinders the Schiff’s base formation between ADA and GEL resulting in an aggregation of particles which disrupts the internal hydrogel structure making it more prone to degradation. It can be therefore concluded that the incorporation of FA and MBGNs has a high impact on the degradation behaviour of ADA-GEL-based films and scaffolds. Especially the combination of both, FA and MBGNs, might be an option to adjust the final degradation of the composite material and thus achieve a more controlled drug release kinetics.^33^

### Drug release studies

FA, Ca and GEL release is described in this section. Especially the release from films for all tested inks was considered. However, as the final application is bioprinting, for the most promising compositions, which were ADA-GEL, ADA-GEL-FA, ADA-GEL-FA-0.1%MBGN and ADA-GEL-FA-0.5%MBGN, release studies using 3D printed scaffolds were additionally carried out. Due to the 3D printing process, the weight of each scaffold is not totally controllable. Therefore, to compare the release from films and scaffolds, the release values of scaffolds were normalized by the average film weight of each composition, respectively. As expected, in all drug release studies a faster release from 3D printed scaffolds was confirmed due to due to higher surface area and due to the faster degradation of scaffolds in comparison to films. A rough estimation of the exposed surface of 3D printed scaffolds compared to films is illustrated in Figure S1 in the supplementary part.

#### FA release

In our previous study^20^ we proved the antibacterial and antioxidant properties of FA within ADA-GEL hydrogels, which might be released from ADA-GEL-based hydrogels. In order to show the impact of MBGN content on the release capability of ADA-GEL and thus a possible obstacle for the FA therapeutic effect, a release study was performed. The cumulative FA release from films and 1 × 1 cm^2^ 3D printed scaffolds with/without MBGNs over an incubation time period of 28 days is illustrated in Figure 5(e–f) and was measured at λ = 310 nm.^59^ After 28 days of incubation the release from ADA-GEL-FA, ADA-GEL-FA-0.1%MBGN and ADA-GEL-FA-0.5%MBGN films reached values of 29.27 ± 2.31 µg/ml, 24.84 ± 2.11 µg/ml and 27.69 ± 3.49 µg/ml, respectively. In comparison, the FA release from ADA-GEL-FA, ADA-GEL-FA-0.1%MBGN and ADA-GEL-FA-0.5%MBGN 3D printed scaffolds reached higher values; up to 288.65 ± 44.56 µg/ml, 226.01 ± 39.17 µg/ml and 275.08 ± 34.19 µg/ml, respectively. The velocity of diffusion proportionally depends on the surface area of samples,^60^ resulting in a higher release for 3D scaffolds with respect to films which exhibit lower surface to volume ratio. One can observe that for both systems (films and 3D printed scaffolds) the FA release is the fastest for neat ADA-GEL-FA samples without MBGNs. The release from ADA-GEL-FA-MBGN films and 3D scaffolds shows a more controlled behaviour, which might be explained with the additional internal crosslinking of ADA as result of the release of Ca ions from MBGNs. It is reported that apart from Ca ions coming from the CaCl_2_ crosslinking solution, the released Ca ions from MBGNs could lead to a slow in situ crosslinking.^61^ This behaviour may cause a more dense hydrogel network resulting in a slower FA release kinetic.^55,61^ A similar deceleration of drug release (icariin was used) due to the presence of inorganic nanofillers compared to the neat reference materials was reported elsewhere.^62^ Another explanation behind the decreased release of FA in the presence of MBGNs might be the hydrophilic structure of MBGNs. The ability to keep more water in the structure may also maintain FA molecules inside the hydrogel slowing down the release.^24^ Moreover, with increasing MBGN content in films and scaffolds a faster degradation is observed. This can be due to the fact that a higher MBGN content within ADA-GEL hydrogels may disturb the Schiff’s base reaction between ADA and GEL leading to a faster FA release. With higher MBGN content a lower degree of crosslinking was determined, which indicates the Schiff’s basedisruption (see section “Degree of crosslinking”). In addition, it is known that the degradation behaviour of the carrier has a significant impact on drug release rate and amount.^33^

#### Ca release

Figure 5(g) illustrates the cumulative Ca release from films prepared with all six hydrogel compositions, whereas the Ca release from 3D printed ADA-GEL, ADA-GEL-FA and ADA-GEL-FA-MBGN scaffolds is illustrated in Figure 5(h). In general, the cumulative Ca release might be derived from the CaCl_2_ used for the crosslinking solution as well as from the Ca content of MBGNs (nominal composition 70%SiO_2_-30%CaO, in mol%), incorporated in the inks. Within the first 3 h, the Ca content is expected to be derived mainly from the CaCl_2_ solution used for crosslinking (see supplementary part Figure S2(a) for films and Figure S2(b) for scaffolds). In Figure 5(g) it is observed that the Ca release from films continuously increased to 0.01 – 0.02 µg/µl for all compositions. No significant difference among all tested hydrogels films could be observed, which might be an indicator for Ca detection coming only from the crosslinking solution. In the first incubation hours Ca ions released by MBGNs most likely act as additional crosslinkers in ADA-GEL and are therefore not detected. As a consequence, these ions might lead to a more dense network and increase the stability of hydrogel films over time. The release from ADA-GEL, ADA-GEL-FA and ADA-GEL-FA-MBGN 3D printed scaffolds is illustrated in Figure 5(h). The release differences can be explained on one hand by the higher MBGN content, which leads to a higher Ca ion concentration in the external medium, and on the other hand by the mentioned accelerated degradation behaviour (see section “In vitro degradation/swelling behaviour”). The degradation of ADA-GEL-FA-0.5%MBGN results in a greater release of Ca. This release behaviour was consistent when comparing the release from films illustrated in Figure 5(g). Even though Ca ions are crucial for various intracellular processes, the control of Ca release is important since a too high content might be toxic and resulting in cell death.^63^ The calcium concentration values recorded from the films were noticeably lower than those reported from 3D printed scaffolds, due to the films lower surface-to-volume ratio when compared to the open-pore scaffolds.^58^ Since calcium is a major inorganic element in native human bone and since calcium regulates the activation of osteoblast cells, the controlled release of Ca ions is therefore crucial for bone TE.^64^

#### GEL release

Figure 5 illustrates the cumulative GEL release from i) films and j) 3D printed scaffolds. The colorimetric experiment is based on the dye binding of Coomassie Brilliant Blue G-250 (Bradford) to proteins like GEL in acidic solution. The colour change, which occurs after adding the Bradford solution to the samples, is the result of interactions between GEL (protein) and the Bradford solution.^65^ The GEL released from films for all compositions (listed in Table 1) showed an initial burst release within the first 3 h (see supplementary part Figure S2(c)). However, with longer incubation time the release remained quite constant. This might be due to various bonding mechanisms present in the hydrogel such as the additional crosslinking between GEL and FA, the internal crosslinking due the Ca ions released from the MBGNs and the Schiff’s base formation.^9,21,55^ Moreover, the reduced release might be a result of an intermolecular renaturation of GEL by interchain hydrogen bonds when the percentage of GEL is high.^33,66^ Lazzara et al.^67^ elucidated this phenomenon, attributing it to a delay in protein release caused by protein adsorption within a palisade matrix, thereby illustrating a sequential process of release, resorption, and re-release.^67^ Interestingly, from incubation day 14 it seems that GEL release is slightly increased for ADA-GEL-0.1%MBGN samples, which might be explained with the lack of additional crosslinking between FA and GEL.^21^ Moreover, the result suggests an absence of interaction between GEL and MBGNs, which might be subsequently compensated through the inclusion of FA.^33^ However, the difference is not significant. In Figure 5(j) an increased GEL release from scaffolds over 28 days of incubation can be seen (see supplementary part Figure S2(d)) for the first 3 h). The release might be explained due to the gradual degradation of scaffolds and the reversible Schiff’s base formation between ADA and GEL.^41^ After 7 days of incubation, GEL release from 3D printed ADA-GEL-FA-0.5%MBGN scaffolds is faster than from ADA-GEL-0.1%MBGN and the reference scaffolds without MBGNs. This result could be due to the fact that the Ca ions released by the MBGNs might increase the formation of the “egg-box” structure between ADA chains, creating a more dense network. This effect can prevent the accessibility of the aldehyde groups for the Schiff’s base reaction with NH_2_-groups of GEL, which are consequently released into the internal medium.^55^ Moreover, the relatively high concentration (0.5% (w/v)) of MBGNs might additionally result in aggregation, which could cause a disruption of the internal network of the hydrogel (breaking of the Schiff’s base formation) facilitating the release of GEL from the scaffolds. This assumption may also explain the faster degradation of ADA-GEL-FA-0.5%MBGN scaffolds and faster FA release from ADA-GEL-FA-0.5%MBGN scaffolds in contrary to ADA-GEL-FA-0.1%MBGN scaffolds. The degree of crosslinking study indicates a decrease of Schiff’s base formation with increased MBGN content. A lower percentage of MBGNs appeared to be more suitable to be incorporated into ADA-GEL-based hydrogels preventing the loss of too much GEL, which is required for cell interaction. Comparing films with scaffolds, an accelerated GEL release from scaffolds is visible due to a higher exposition of the surface of scaffolds. Monavari et al.^33^ investigated the GEL release from ADA-GEL 3D printed scaffolds with bioactive glass nanoparticles with/without icariin. In their work a constant release of GEL during 35 days of incubation was shown reaching values of 4 mg/mL to 7 mg/mL.^33^ Their GEL release from scaffolds was in a higher range than in the present study, which might be due to the additional enzymatic crosslinking with mTG and the additional crosslinking of FA to GEL present in our study.^15,21^ Moreover, larger scaffolds were printed in their work, resulting in more material present and hence higher GEL release.^33^

### Degree of crosslinking

In our previous work the impact of FA on the Schiff’s base formation in ADA-GEL was already shown^20^. It is apparent that the highest concentration of 0.5% (w/v) MBGNs within ADA-GEL-based hydrogels might disrupt the hydrogel network leading to interruption of the Schiff’s base formation resulting in a faster degradation of samples, faster FA release and accelerated GEL release, indicating that a degree of crosslinking test is of the highest importance. To determine the effect of MBGNs on the reversible crosslinking of ADA and GEL, pure GEL was used a reference. Figure 7(f) shows the highest degree of crosslinking (18%) for ADA-GEL-FA samples confirming that FA crosslinks to GEL resulting in less free NH_2_-groups and thus higher degree of crosslinking. The ADA-GEL degree of crosslinking was 13%. ADA-GEL and ADA-GEL-0.1%MBGN reached the same range of crosslinking degree, indicating that a concentration of 0.1% (w/v) MBGNs does not hinder or interrupt the Schiff’s base formation. ADA-GEL-FA-0.1%MBGN samples reached slightly higher degree of crosslinking (15%), which might be again an indication of the additional crosslinking between FA and GEL, however, not significantly different. Interesting is that ADA-GEL-0.5%MBGN reached the lowest degree of crosslinking with 11% indicating that a high MBGNs content might disrupt the hydrogel network resulting in a disruption of Schiff’s base formation and thus a lower degree of crosslinking. Comparing ADA-GEL-0.5%MBGN with ADA-GEL-FA-0.5%MBGN one can observe a higher degree of crosslinking for FA containing samples which again confirms the additional crosslinking of FA and GEL. ADA-GEL-FA and ADA-GEL-FA-0.1%MBGN samples appeared to have the highest degree of crosslinking, especially notable during 3D printing, resulting in high resolution and high shape fidelity of 3D printed scaffolds with these two inks (see 3D printing section).

### HA formation

**Figure 6.**
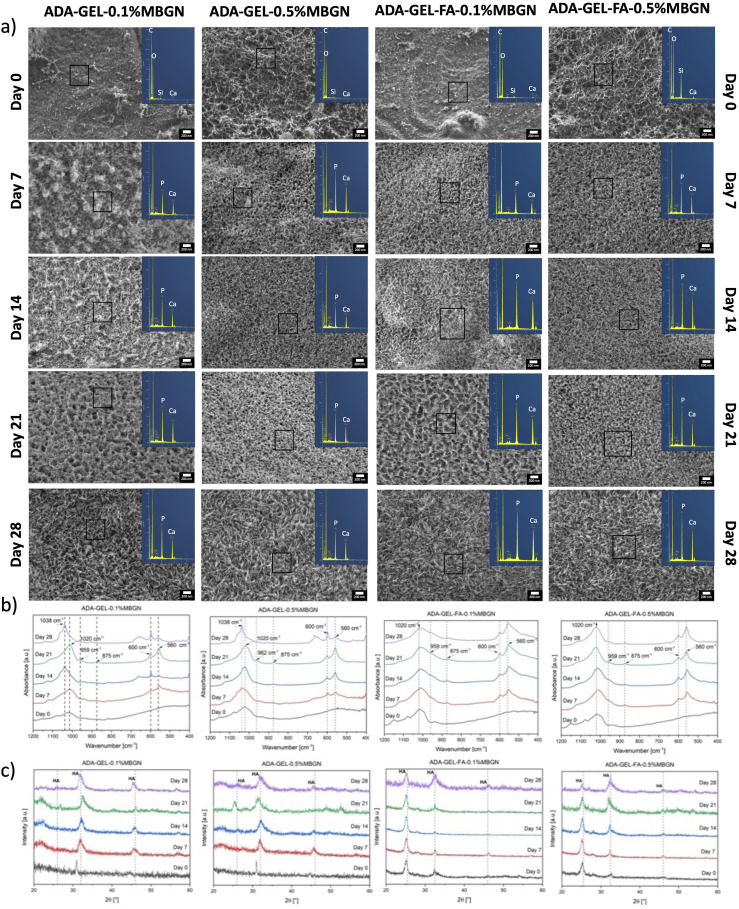
Confirmation of the formation of HAp on the surface of ADA-GEL-0.1%MBGN, ADA-GEL-0.5%MBGN, ADA-GEL-FA-0.1%MBGN and ADA-GEL-0.5%MBGN samples after being immersed in SBF for 28 days with (a) SEM-EDS analysis (scale bar: 200 nm), (b) ART-FTIR analysis and (c) XRD patterns.

The formation of a HAp layer on the surface of MBGN containing samples demonstrates their bioactive nature and their potential for promoting osteoconductivity.^68^ To confirm the bioactivity of MBGNs and investigate the impact of FA on this property, a bioactivity study was performed. Briefly, the hydrogel films of ADA-GEL-0.1%MBGN, ADA-GEL-0.5%MBGN, ADA-GEL-FA-0.1%MBGN and ADA-GEL-FA-0.5%MBGN were immersed in SBF for 28 days. The confirmation of HAp formation was analysed by SEM-EDS (Figure 6(a)), ART-FTIR (Figure 6(b)) and XRD (Figure 6(c)) analyses. SEM-EDS analysis was used to visualize the ADA-GEL composite films and determine the elemental composition of HAp. After 28 days the elemental characterization through EDS analysis confirmed the formation of HAp for ADA-GEL-0.1%MBGN with Ca/P = 1.32 (atomic%), for ADA-GEL-0.5% with Ca/P = 1.36 (atomic%), for ADA-GEL-FA-0.1%MBGN with Ca/P = 1.54 (atomic%) and ADA-GEL-FA-0.5%MBGN with Ca/P = 1.56 (atomic%). According to literature HAp with a calcium deficiency, denoted as Ca_10−x_ (PO_4_)_6_−x (HPO_4_)x (OH)_2−x_, where 0 ≤ x ≤ 1, is of higher biological significance compared to stoichiometric HAp. This interest arises from the fact that the Ca/P ratio in bone closely approximates 1.5.^69–71^ The ideal Ca/P ratio in atomic% for HAp with the composition Ca(PO_4_)_3_OH is 1.67. This ideal ratio is rather difficult to achieve, since this value refer to a EDX measurement with a flat surface, which might not be the case for ADA-GEL samples after critical point drying. Moreover, due to the presence of phosphor (P) in the SBF solution the actual P content might be higher resulting in a deficiency of Ca ions. Therefore, in this study, the EDX analysis should simply give a rough overview about the presence of Ca and P. To identify the formation of HAp on the surface of the sample more precisely FTIR and XRD measurements were performed. In Figure 6(b), in all tested compositions sharp peaks at 1038 cm^−1^ or 1020 cm^−1^ are visible, which indicate PO_4_^3-^ stretching and is therefore an indication for HAp. This peak becomes more prominent after 14 days of incubation in SBF.^68,72^ The peak at 875 cm^−1^ stands for CO_3_^2-^ vibration.^72^ Moreover, the shoulders at 875 cm^−1^ and 959 cm^−1^ might be associated with HAp.^68,72^ In all compositions, peaks at 560 cm^−1^ and 600 cm^−1^ were evident and can be associated to the P-O bond in crystallized calcium phosphate, indicating the formation of a CaP phase.^9,72,73^ All mentioned peaks are summarized in Table 2. Figure 6(c) illustrates that for all compositions, the main three patterns for HAp appear at 2θ = 26°, 32° and 46°, that are attributed to (002), (211), and (310) lattice planes in the HAp crystals, which is also a confirmation of the bioactivity of MBGNs and HAp formation.^33,48,68,74^ Besides, it can be concluded, that even though FA appeared to stabilize the films due to additional crosslinking to GEL within the first 7 days (as mentioned above), it does not hinder the contact of MBGNs with SBF and the consequent formation of a HAp layer on the surface from day 7, as it was for films without FA. Therefore, FA does not have a negative impact on the bioactivity of ADA-GEL-MBGN samples. Thus, with ADA-GEL-FA-MBGN hydrogels, constructs with increased stability and bioactive properties were created.

**Table 2.** Summary of FTIR peaks (see Figure 6(b) and (c))

Vibration	Wavenumber (cm^−1^)	Refs
Stretching mode of PO_4_^3-^	1038, 1020	^68,72^
Vibration of HPO_4_^2-^	875, 959	^68,72^
Bending mode of CO_3_^2-^ vibration	875	^72^
Bending mode of PO_4_^3-^ vibration	560, 600	^9,72,73^

To additionally confirm the bioactive properties of the MBGNs, pure 0.1% (w/v) and 0.5% (w/v) MBGNs were immersed in SBF solution for 7, 14 and 21 days of incubation. The Si and Ca ion release was measured with ICP-OES (results shown in supplementary part in Figure S3). However, a slightly lower Si ion release was measured compared to Ca. This behaviour might be explained due to the fact that Ca is loosely bonded in the silicate network. Si ions are released slowly and in a sustained manner. Due to the high surface area of MBGNs, initially a fast reaction occurred at the interface which consequently leaves to the precipitation of the calcium phosphate layer. This CaP layer can also act as a barrier to the faster release of Si ions. Moreover, the lower Si release might be explained due to the supersaturation of Si-ions in the solution. Due to the high surface area of the particles, initially a quick reaction (ion exchange) between SBF and MBGNs occurs which results in formation of the HAp layer. This HAp layer can restrict or slow down the particle degradation, which can be seen by the low release of Si ions.

### Mechanical characterization

#### Compression test

**Figure 7.**
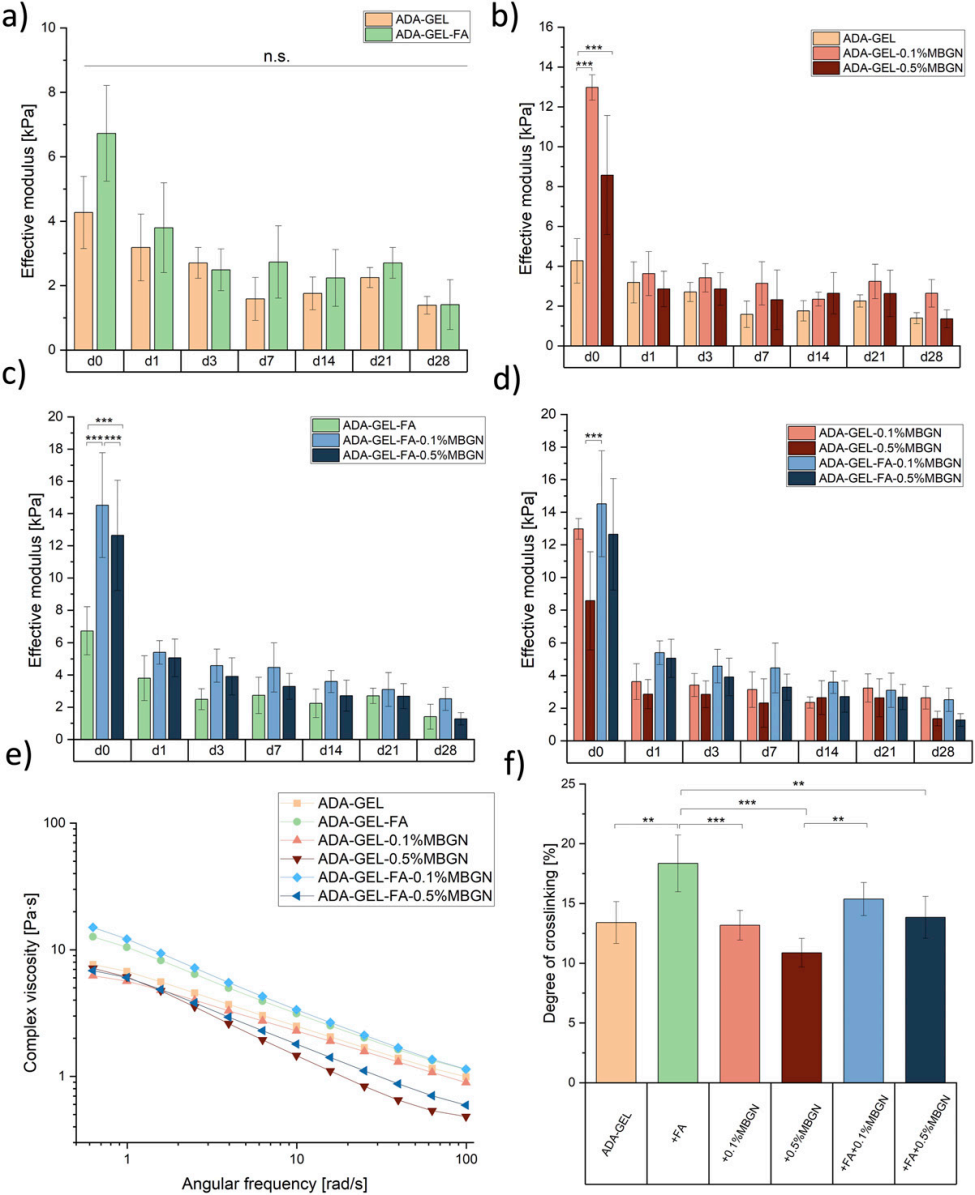
Comparison of the effective modulus of: (a) ADA-GEL and ADA-GEL-FA hydrogel samples, (b) ADA-GEL-0.1%MBGN and ADA-GEL-0.5%MBGN hydrogel with ADA-GEL as reference and (c) ADA-GEL-FA-0.1%MBGN and ADA-GEL-FA-0.5%MBGN hydrogel with ADA-GEL-FA as reference. (d) ADA-GEL-0.1%MBGN, ADA-GEL-0.5%MBGN, ADA-GEL-FA-0.1%MBGN and ADA-GEL-FA-0.5%MBGN hydrogel. All samples were incubated over 28 days of incubation in cell culture medium. (e) Complex viscosities of all compositions using the frequency sweep. (f) Degree of crosslinking. Statistical analysis was performed via one-way ANOVA using the Bonferroni test with *p* < 0.05 = *, *p* < 0.01 = ** and *p* < 0.001 = ***. n.s. = not significant different.

One aim of this study was to determine the capability of FA and MBGNs to increase the mechanical strength of hydrogels. Especially, due to the fact that FA has been reported to induce further bonding with the polymer matrix, leading to an increase of mechanical strength of hydrogels.^75,76^ Moreover, incorporation of MBGNs should improve the mechanical properties of hydrogels.^33,77^ For this reason, the measurement of the effective modulus is crucial and was performed for all used hydrogels (listed in Table 1) in compression using a Microtester. Figure 7(a)–(d) illustrates the effective modus of all compositions. To ensure a better comparison of the compositions among each other and to improve the visualization of the FA and MBGN impact on the effective modulus, results are presented in different graphs. In Figure 7(a) a clear trend is visible that for certain time points a higher effective modulus was measured for ADA-GEL-FA samples, which might be explained by the additional crosslinking of FA to GEL.^21^ However, the results are not significantly different. In Figure 7(b) ADA-GEL-0.1%MBGN and ADA-GEL-0.5%MBGN samples are compared using neat ADA-GEL samples as reference material. At each time point an increase of the effective modulus for MBGN containing samples is seen. This result can be explained due to the Ca ion release from MBGNs, which leads to a more stabilized ADA-GEL matrix. Interestingly, the compositions with lower MBGN content (0.1% (w/v)) seem to exhibit a higher effective modulus than ADA-GEL-0.5%MBGN samples. This result could confirm the assumption that agglomerates of MBGNs at high concentrations could disrupt the internal matrix of ADA-GEL hydrogel, leading to lower mechanical properties. Similar behaviour is visible in Figure 7(c) where ADA-GEL-FA-MBGN samples were compared using ADA-GEL-FA as a reference material. In this comparison ADA-GEL-FA-0.1%MBGN samples show the highest effective modulus measured during the entire 28 days of incubation compared to pure ADA-GEL-FA samples, which might be again explained by the additional crosslinking with Ca ions released from MBGNs. The increase in stiffness due to the presence of inorganic fillers was already reported by Wei et al.^78^ and Marelli et al.,^79^ which studied the mechanical properties of collagen hydrogels loaded with nanosized BG particles incubated in SBF. They explained their increase of stiffness with the mineralization behaviour due to incubation in SBF solution. However, since in our study films were immersed in cell culture medium the results are difficult to compare. On the other hand, ADA-GEL-FA-0.5%MBGN samples show a lower effective modulus compared to ADA-GEL-FA-0.1%MBGN samples, which most likely refer to the disruption of the entire hydrogel network with higher particle concentration. A similar result was found by Heid et al.^80^ who investigated the mechanical properties of ADA-GEL hydrogels loaded with 0.1% and 0.5% (w/v) bioactive inorganic fillers (BIF). During the entire incubation time of 21 days, the modulus values ranged between 3.7 and 6.5 kPa showing higher moduli for samples with lower 0.1% (w/v) MBGN content compared to 0.5% (w/v) MBGN loaded samples. They explained this behaviour with an unpredictable location of particles between the covalent bonding of ADA and GEL leading to a disruption of the Schiff’s base formation and thus a decrease of internal stability of the ADA-GEL-based samples which might be an additional explanation for the present results and similar mechanical behaviour.^80^ Comparing pure ADA-GEL-MBGN samples with ADA-GEL-FA-MBGN samples in Figure 7(d) for all measured time points one can observe an increased effective modulus for samples containing FA compared to pure MBGN samples. Moreover, a decrease of effective modulus for 0.5% (w/v) compositions was measured indicating the disruption of the hydrogel network due to high MBGN content. However, this behaviour becomes not significantly different with increasing incubation time. Another reason for the decreased mechanical stability of ADA-GEL-based samples with high 0.5% (w/v) MBGN content is likely related to the accelerated degradation mentioned above compared to samples with 0.1% (w/v) MBGNs. However, it should be mentioned that for compression tests small films were used compared to the samples used for the degradation study, which might affect the degradation behaviour and thus explain the decrease of mechanical stability of all compositions. The volume to surface ratio is higher for samples used for compression testing leading to a larger surface exposed to the surrounding cell culture medium. Observing all compositions, the effective modulus of the hydrogels decreases after being immersed in cell culture medium from day 1, however, it maintained a relatively constant mechanical stability over the total incubation period of 28 days. Especially ADA-GEL and ADA-GEL-FA samples do not show a significant decrease of mechanical properties after immersion, while the effective modulus of the other compositions (with MBGNs) drops significantly. Additionally, this result might be explained with the hydrophilic behaviour of MBGNs, which can absorb water after being immersed, thus leading to an accelerated decrease of mechanical properties.^24^ Distler et al.^14^ for instance investigated ADA-GEL hydrogels crosslinked with CaCl_2_-solutions with a variation of mTG concentrations. Considering their effective modulus for samples crosslinked with a 2.5% (w/v) mTG solution, as it was done in our study, they reported values in a comparable range of 5 – 10 kPa. Moreover, they mentioned that a stiffness of up to 120 kPa might be achieved by increasing the mTG concentration to 10% (w/v).^14^ Our measured effective moduli are also comparable with those reported by Sarker et al.,^81^ who measured a reduced Young’s modulus for comparable ADA-GEL hydrogels (2.5%ADA and 2.5%GEL in 1:1 ratio) of ∼10 kPa before incubation. Even though an increase of effective modulus of the hydrogels due to the presence of FA and MBGNs is measured in our study, the mechanical properties of the present scaffolds may not be enough for them to support loads in the context of bone regeneration. On the other hand, the effective modulus in compression revealed application potential in contact with soft tissues such as kidney (5-10 kPa),^82^ liver (1-6.5 kPa),^83^ cardiac muscle (up to 8 kPa),^84^ or cartilage (15-20 kPa).^85^ Depending on the used cell type the substrate stiffness for cell growth and proliferation should fulfil certain requirements. Due to the MBGN content in our samples, the material is in principle intended for bone TE applications. Therefore, for the direct cell study (see section “Direct test”) and 3D bioprinting study (see section “3D bioprinting”) the preosteoblastic cell line MC3T3-E1 was used to investigate the cell behaviour. As reported by Distler et al.^14^, it is possible to increase the mechanical stiffness by increasing mTG and CaCl_2_ concentration which might be considered for future studies.

#### Rheological evaluation

The rheological properties of ADA-GEL-based hydrogels were analysed to assess their 3D printability. The strain rate for amplitude sweep was set at 10% based on a previous parameter identification test.^20^ Therefore, for the present study a constant elongation oscillation strain of 10% was used to measure the frequency sweep test of FA and/or MBGN containing ADA-GEL hydrogels for all used inks (listed in Table 1). In Figure 7(e) it is shown that the complex viscosity decreases with increasing angular frequency for all used inks. This behaviour is characteristic for the shear thinning behaviour of materials.^86^ This property is crucial since shear thinning allows materials to be injected using a shear force and they regain their initial structure after removal of that force. This behaviour allows them to be extruded to create 3D printed structures.^87^ At 1 rad/s the viscosity is between 6 and 12 Pa∙s, whereas the highest complex viscosity was measured for ADA-GEL-FA-0.1%MBGN inks and the lowest for ADA-GEL-0.5%MBGN. With increasing angular frequencies for compositions with the highest MBGN content, ADA-GEL-0.5%MBGN and ADA-GEL-FA-0.5%MBGN, the lowest viscosities (0.4 Pa∙s and 0.5 Pa∙s) were measured, respectively. This phenomenon might be explained by the fact that a higher amount of MBGNs could disturb the internal Schiff’s base formation between ADA and GEL leading to a lower viscosity of the hydrogel. These results correlate with the observations reported in section “Degree of crosslinking”, where a decreasing degree of crosslinking with increasing MBGN content was shown. On the other hand, the addition of 0.1% (w/v) MBGNs and FA seems to stabilize the inks resulting in the highest measured viscosities for ADA-GEL-FA and ADA-GEL-FA-0.1%MBGN inks. This result might be explained by the additional internal crosslinking due to the released Ca ions from MBGNs and the additional crosslinking between FA and GEL.^21,55^ The additional internal crosslinking is advantageous to ensure a higher resolution of 3D printed scaffolds. Interestingly, Zhu et al.^88^ evaluated the rheological properties of ADA-GEL hydrogels loaded with both MBGNs and amine-functionalized MBGNs (AMBGN). The functionalization of MBGNs with NH_2_-groups was implemented to establish additional bonding with GEL chains. This additional bonding of GEL is comparable with what was aimed at in this work with FA. In their study, the functionalized particles showed an increased viscosity in comparison to the control material, however, a concentration of 1% (w/v) MBGNs also led to a decrease in viscosity and effective modulus. Additionally, Heid et al.^80^ also reported a decrease in complex viscosity with the incorporation of 0.5% (w/v) bioactive inorganic fillers (BIFs) compared to the composition with 0.1% (w/v) BIFs.^80^ A decrease of viscosity and effective modulus (see section “Compression test”) with increasing MBGN content was also confirmed in our study. Considering the rheological results, it might be concluded that the most suitable hydrogels for 3D printing are ADA-GEL-FA and ADA-GEL-FA-0.1%MBGN, which will be further discussed in the next section. 

### Assessment of 3D printability

The 3D printability was assessed using different tests, named FFT, FCT, and GST. This evaluation aimed to comprehend the qualitative and quantitative effects of FA and MBGNs on the resolution, shape fidelity, and cohesion of extruded 3D printed ADA-GEL scaffolds. The outcomes of these tests were utilized to select the most appropriate inks. Subsequently, the optimal inks were employed for a more detailed 3D printing assessment.

#### FFT

**Figure 8.**
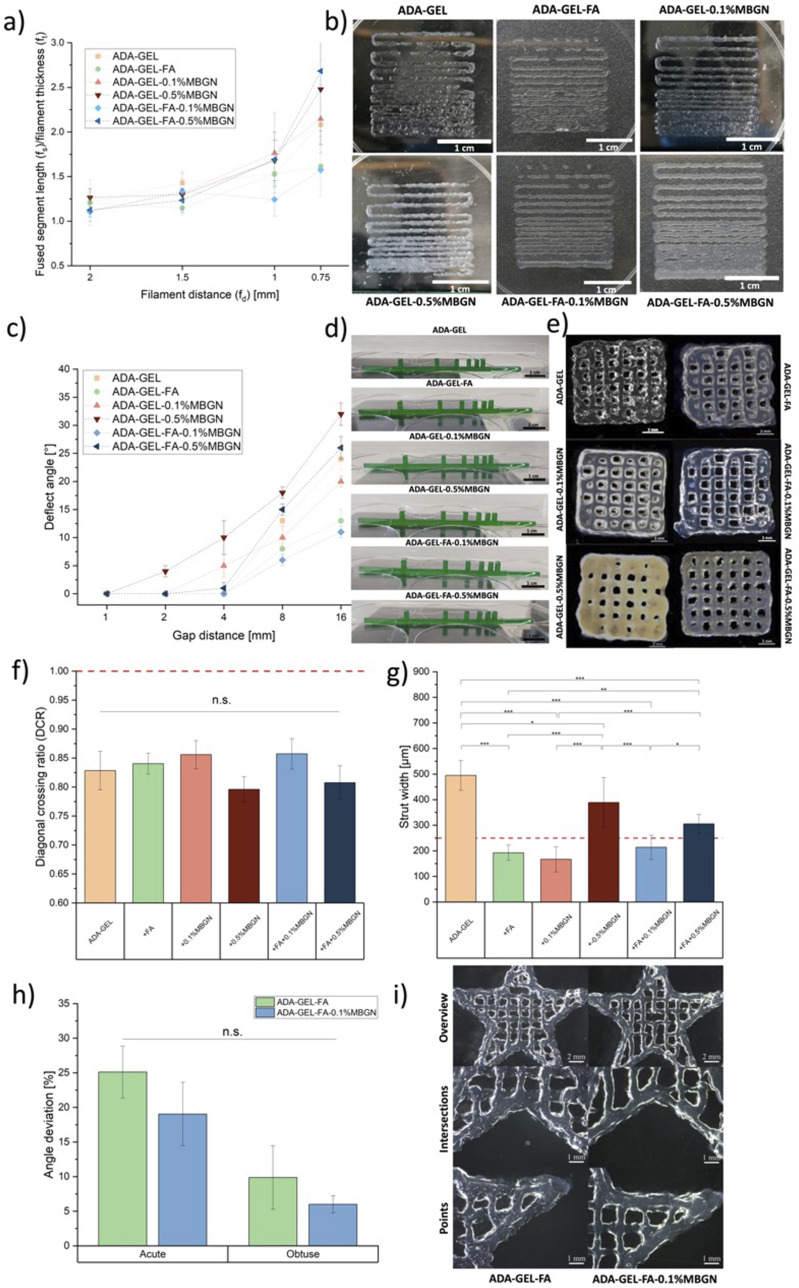
(a) FFT with five different strut distances, ranging from 2 mm to 0.5 mm. (b) Photos of structures printed for FFT. (c) Deflection angle of the FCT test vs gap distance. (d) Photos of the strands. Scale bar: 1 cm. (e) Light microscopy images of all printed ADA-GEL-based inks. Scale bar: 2 mm. (f) DCR and (g) strut width of all compositions. (h) Angular deviation for acute and obtuse angles of ADA-GEL-FA and ADA-GEL-FA-0.1%MBGN. (i) Light microscopy images of star-shaped structures. Scale bar: 1 mm and 2 mm. Statistical analysis was performed via one-way ANOVA using the Bonferroni test with *p* < 0.05 = *, *p* < 0.01 = ** and *p* < 0.001 = ***.

In Figure 8(a), the correlation between the fused segment length (f_s_) and filament thickness (f_t_) (f_s_/f_t_) is plotted against the filament distance (f_d_). The photos used for evaluating this test are shown in Figure 8(b). As the ratio (f_s_/f_t_) approaches 1, there is a higher probability that the strains will exhibit a square geometry along the edges while maintaining consistent thicknesses. This suggests a steady printing of segments.^87^ However, it was not possible to achieve a 0.5 mm filament distance resolution for the printed inks due to the merging of strains. For all inks, an increase in filament distance from 1.5 mm to 2 mm corresponds to a higher resolution, resulting in an f_s_/f_t_ ratio close to 1. Conversely, when distances decrease from 1 mm to 0.75 mm, fluctuations indicate strands fusing together, leading to an increase of the f_s_/f_t_ ratio. At small distances, ADA-GEL-FA and ADA-GEL-FA-0.1%MBGN tend to be closer to 1 compared to other inks, indicating their suitability for 3D printing. This behaviour aligns with the viscosity measurements discussed in the section “Rheological evaluation”, showing the highest viscosity for these two inks and this may be the reason for the obtained best-printed pattern. Moreover, the enhanced printability can be explained by the additional crosslinking of FA and GEL.^21^ The release of Ca ions from MBGNs seems to lead to a more stable printing, as reported elsewhere.^80^ However, samples with high MBGN content in ADA-GEL-0.5%MBGN and ADA-GEL-FA-0.5%MBGN show low-resolution printing, indicated by a higher f_s_/f_t_ ratio at small distances. This result might be explained by the decreased viscosity of these inks mentioned above, and the assumption that 0.5% MBGN content might disrupt the hydrogel network, leading to increased fusion of strands during printing. Summarizing, FFT indicated the suitability of ADA-GEL-FA and ADA-GEL-FA-0.1%MBGN inks for 3D printing.

#### FCT

The FCT is used to assess the maximum distance between two objects, over which a printed strand does not collapse. This is particularly crucial for porous structures to prevent the tearing down of strands over pores. For the evaluation of the deflection angle, photos of printed strands on top of the pillars were used, as shown in Figure 8(d). The relationship between the deflection angle and pillar distances is illustrated in Figure 8(c). It is evident that, across all printed compositions, the deflection increases as the distance between pillars increases. For ADA-GEL-0.5%MBGN, even at a small gap distance of 2 mm, a deflection angle of 4 ± 1° could be measured, possibly due to the increased weight of inks attributed to the higher content of MBGNs resulting in a fast collapse of strands. Especially with increasing gap distances, this phenomenon is more visible for ADA-GEL-0.5%MBGN and ADA-GEL-FA-0.5%MBGN inks, reaching values of 11 ± 1° and 26 ± 2°, respectively. This is followed by ADA-GEL and ADA-GEL-0.1%MBGN inks. However, ADA-GEL-0.1%MBGN shows lower values compared to pure ADA-GEL inks, indicating the beneficial addition of low MBGN content due to the inner crosslinking by Ca ions released by MBGNs.^80^ Overall, ADA-GEL-FA and ADA-GEL-FA-0.1%MBGN inks show the smallest deflection angle with increasing gap distances, highlighting them as the most suitable inks. The improved printability of these two inks can be attributed to the additional crosslinking of FA and GEL^21^ and the presence of Ca ions released by MBGNs^80^ mentioned above. Moreover, the increased viscosity and increased degree of crosslinking (see section “Degree of crosslinking”) of these two inks can also contribute to the improved printing properties compared to other inks.

#### GST

After assessing the best resolution of inks (FFT) and the ability to create porous structures (FCT), 3D printed grids were generated in terms of a GST for a quantitative evaluation of shape fidelity and geometry accuracy in 3D printing. The diagonal crossing ratio (DCR) served as an indicator to assess the pores of 3D printed scaffolds, determining whether the strands maintained their ideal pore shape or fused when stacked.^43^ Hazur et al.^43^ considered 354 µm as an ideal diagonal while printing 1 × 1 cm^2^ grids with strands of 250 µm. In this study, the ideal diagonal (d_ideal_) of a pore was calculated by measuring the pore width and height (see Figure 3(c), w_v,x_, w_h,x_, x = 5 replicates). The measurement was repeated five times within one light microscope image of a 3D printed scaffold. The light microscopy images used for the evaluation of pores and struts are displayed in Figure 8(e). According to equation (4), d_ideal_ was calculated. The actual diagonal (d_m_) of the evaluated pores was also measured with ImageJ. Finally, the DCR was determined according to equation (5) as the quotient between d_m_ and d_ideal_, as illustrated in Figure 8(f). The ideal DCR value was 1.0, with a decrease in value towards zero indicating a rounder pore. It is observed that ADA-GEL-FA, ADA-GEL-0.1%MBGN, and ADA-GEL-FA-0.1%MBGN inks exhibited the closest values, while samples containing 0.5% (w/v) MBGNs led to a decreased value resulting in strand fusion. The stabilization of pores in the former inks may be attributed to the additional crosslinking of FA and GEL^21^ and the release of Ca ions from MBGNs within the inks.^80^ However, an excessively high content of MBGNs leads to the opposite effect. Similar improvements in 3D printing shape fidelity were confirmed by the related tests (FFT and FCT) discussed above. For the GST, a nozzle with a diameter of 250 µm was used, considering the ideal strut size as 250 µm. To evaluate the struts, five different strut widths vertically and horizontally (see Figure 3(c), s_v,x_, s_h,x_, x = 5 replicates) on one light microscopy picture were measured. In Figure 8(g), the mean of the measured struts is shown, with the ideal strut of 250 µm width marked with a red line. 3D scaffolds printed with ADA-GEL-FA, ADA-GEL-0.1%MBGN, and ADA-GEL-FA-0.1%MBGN inks could be printed with struts closer to 250 µm compared to the other inks. This behaviour correlated with results established for the DCR analysis, again confirming ADA-GEL-FA and ADA-GEL-FA-0.1%MBGNs as the most suitable (printable) inks.

#### Complex star-shape printing

In FFT, FCT, and GST, it was indicated that ADA-GEL-FA and ADA-GEL-FA-0.1%MBGN inks appeared to be the most suitable for 3D printing. To test this finding, these two inks were selected for fabricating more complex and intricate structures. Star shapes were printed to qualitatively and quantitatively assess the reproducibility of edges and vertices. Figure 8(i) shows microscopy images of the entire star (upper images, scale bar: 2 mm), of points of the star (lower images, scale bar: 1 mm), and of the intersections between the points (middle images, scale bar: 1 mm) printed with ADA-GEL-FA and ADA-GEL-FA-0.1%MBGN. In Figure 8(h), the results for angle deviation in percentage, calculated according to equation (6), are displayed. The experimental acute and obtuse angle ( $\theta_{e}$ ) was measured by connecting the vertex to the intersection, as illustrated in Figure 3(d). The results depicted in Figure 8(h) reveal that the ADA-GEL-FA printed structures exhibits a higher deviation from the theoretical angle compared to ADA-GEL-FA-0.1%MBGN ink. This trend underlines the positive impact of Ca ions release into the polymer network.^80^ However, the difference is not significant. Considering the positive trend established in this printing experiment, ADA-GEL-FA-0.1%MBGN ink was used to print larger grids of 1.5 × 1.5 cm^2^ with 1, 4, and 8 layered scaffolds. A bone shape structure was also printed. Results are displayed in Figure S4 (see supplementary part). Successful printing of a bone scaffold (Figure S4(a)) and successful printing up to 8 layers (Figure S4(b)) can be observed. Figure S4(c) additionally shows SEM images of a 4-layer scaffold in 3 different magnifications. Moreover, excellent handling of scaffolds was confirmed after crosslinking the samples with a crosslinking solution composed of 0.1 M CaCl_2_ and 2.5% (w/v) mTG.

In conclusion, the incorporation of FA and 0.1%MBGNs into ADA-GEL inks leads to an increased viscosity attributed to Ca ions released by MBGNs^80^ and the additional crosslinking of GEL and FA.^21^ Furthermore, the rising degree of crosslinking and, consequently, an increased formation of Schiff’s base, could contribute to enhanced printability.

### *In vitro* cytocompatibility assessment

#### Direct test

**Figure 9.**
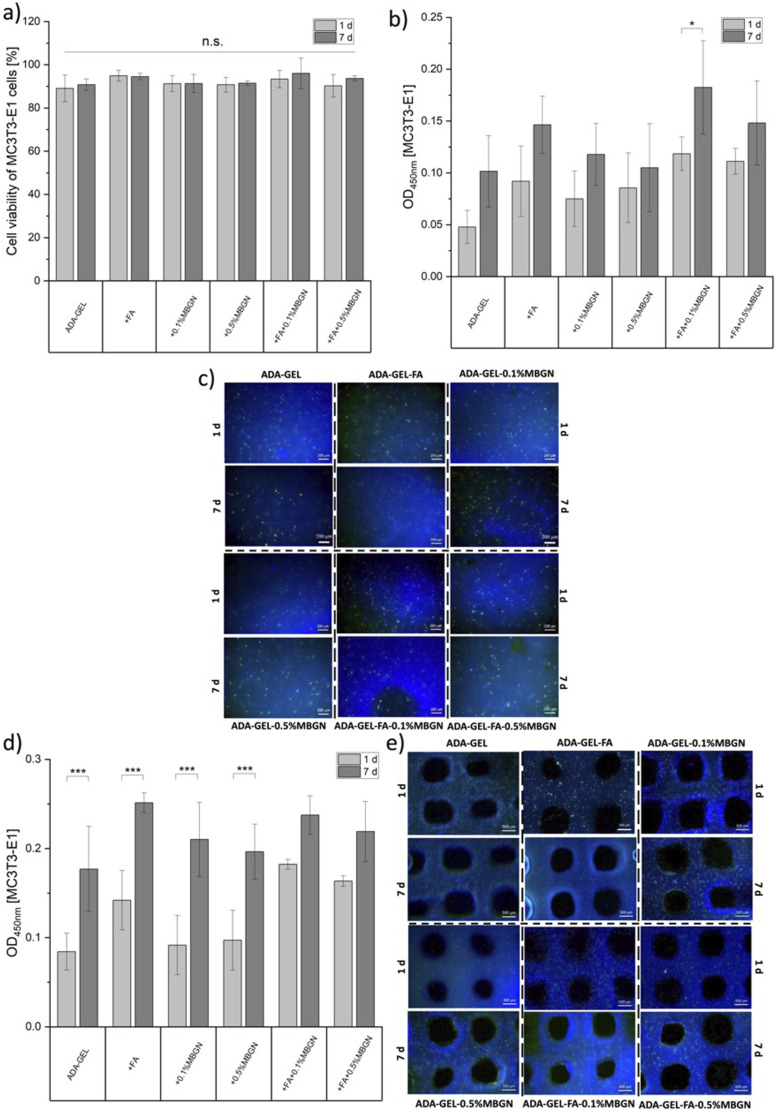
(a) MC3T3-E1 cell viability during direct cell study and cell activities of embedded cells into all bioinks after (b) direct cell study and (d) 3D bioprinting within 24 h and 7 days of incubation. Fluorescence microscopy images of the cells after (c) direct cell study and (e) 3D bioprinting, including ADA-GEL as a control. Green: Calcein AM, blue: DAPI. Scale bar 200 μm and 500 µm. Statistical analysis was performed via one-way ANOVA using the Bonferroni test with *p* < 0.05 = *, *p* < 0.01 = ** and *p* < 0.001 = ***.

In order to assess whether the materials provide a suitable environment for cells, MC3T3-E1 cells were directly incorporated into all hydrogels (compositions listed in Table 1) for 24 h and 7 days of incubation. After certain time points the cell activity was analysed by a WST-8 assay for 4 h. Subsequently, the cell viability was investigated by a Calcein AM and DAPI staining. The medium during incubation time was changed twice a week. Figure 9(b) shows the WST-8 results (optical densities at 450 nm). After 24 h of incubation, MC3T3-E1 cells showed the lowest cell activity of 0.05 within ADA-GEL samples, while they reached the highest cell activity of 0.12 when incorporated in ADA-GEL-FA-0.1%MBGN samples. After 7 days of incubation an increase of activity is visible for all samples, ranging from 0.1 for ADA-GEL up to 0.18 for ADA-GEL-FA-0.1%MBGN samples. Zehnder et al.^89^ reported the cell activity of the MG-63 (osteoblast-like) cell line incorporated into ADA-GEL samples, which was around 0.05. However, 2.5%ADA and 2.5%GEL (w/v) concentrations were used in that study, which might explain the higher cell activity reported in our study in which a higher GEL concentration (3.75% (w/v)) was used. The presence of a higher GEL concentration provides more RGD sequences for cell attachment likely resulting in higher cell activities. The increase of cell activity with increasing incubation time might also be explained with the gradual degradation of the hydrogels during the first week, as discussed in section “In vitro degradation/swelling behaviour”). The degradation leads to higher hydrogel network porosity resulting in better cell migration and proliferation inside the hydrogels.^53^ Since the lowest activity was measured for ADA-GEL samples, the addition of FA and MBGNs appeared to be beneficial for MC3T3-E1 cells. The positive effect of FA on cell behaviour has been already reported in previous studies.^90,91^ The increase of cell activity due to the presence of MBGNs was also confirmed for MC3T3-E1 cells by Monavari et al.^33^ and for mouse dermal fibroblast cells by Wei et al.^78^ Moreover, a positive effect of MBGNs on bone cell activity has been reported by Wu et al.,^92^ who studied MG-63 cells encapsuled in a MBGN/sodium alginate/gelatin hydrogel.^92^ It is notable that higher cell activities are reported for samples with lower 0.1% (w/v) MBGN content compared to samples with higher 0.5% (w/v) MBGN concentration for both measured time points. This result might be explained by the rapid ion release, which might increase the pH of the local environment.^93^ Comparable results were reported by Ye et al.^94^ when examining the activity of bone marrow-derived mesenchymal stem cells (BMSCs) within an alginate and gelatin hydrogel loaded with MBGNs at varying concentrations. Specifically, they observed a decline in cell activity at concentrations exceeding 10% (w/v) after 7 days of incubation, attributed to the alkaline environment created by the MBGNs. However, this difference was not evident after 21 days, suggesting a long-term benefit of the presence of MBGNs in cell cultures.^94^ The highest activity of MC3T3-E1 cells within ADA-GEL-FA-0.1%MBGN samples indicated the most promising composition owing to its favourable combination of FA and a low concentration of 0.1% (w/v) MBGNs. Following the WST-8 assay a Calcein AM and DAPI staining was performed and visualized by taking fluorescence images, as illustrated in Figure 9(c) after 24 h and 7 days of incubation. In all images a high cell distribution and high cell density can be observed. In Figure 9(a) the viability of MC3T3-E1 cells is shown, which was calculated by considering the ratio between alive cells (Calcein AM staining, green dots in Figure 9(c)) and the total amount of cells (DAPI staining, blue dots in Figure 9(c)). For all samples a high MC3T3-E1 cell viability within all hydrogel compositions was determined. After 7 days of incubation a slightly increase of cell viability for ADA-GEL-FA and ADA-GEL-FA-0.1%MBGN samples (95% and 96%, respectively) compared to neat ADA-GEL samples (91%) can be seen but these values are not significantly different. It can be concluded that ADA-GEL-FA and ADA-GEL-FA-0.1%MBGN hydrogels are the most promising inks for MC3T3-E1 cells after 7 days of incubation.

#### 3D bioprinting

To assess the impact of shear stress during 3D printing on MC3T3-E1 cells, a 3D bioprinting test was performed. The MC3T3-E1 cell activity within 3D printed hydrogel scaffolds was measured with a WST-8 assay after 24 h and 7 days of incubation. In Figure 9(d) the optical densities measured at 450 nm are displayed, whereas in Figure 9(e) fluorescence microscopy images of incorporated MC3T3-E1 cells within 3D printed scaffolds of all compositions (Table 1) are shown. In all fluorescence microscopy images, a homogeneous cell distribution and high cell density can be observed. In Figure 9(d) for all samples an increase in cell activity with increasing incubation time was measured. After 7 days of incubation, the highest cell activities for 3D printed MC3T3-E1 cells within hydrogels were measured for ADA-GEL-FA and ADA-GEL-FA-0.1%MBGN scaffolds with 0.25 ± 0.01 and 0.24 ± 0.02, respectively. For bioinks without FA, ADA-GEL, ADA-GEL-0.1%MBGN and ADA-GEL-0.5%MBGN, lower cell activates were measured with 0.18 ± 0.05, 0.21 ± 0.04 and 0.20 ± 0.03, respectively, indicating the positive effect of FA after 7 days of incubation. The increase of cell activity due to the presence of FA and MBGNs was already confirmed during the direct cell study (see above). Especially based on the anti-inflammatory properties of FA^95^ and the cell stimulating properties of MBGNs^96–98^ the high cell activities can be explained. Moreover, the increase of cell activities with increasing incubation time can be explained by the degradation of the 3D printed scaffolds (discussed in section “In vitro degradation/swelling behaviour”) resulting in an accelerated contact to WST-8 solution and thus improved metabolism of MC3T3-E1 cells leading to higher measured optical densities. Moreover, degradation creates a higher porosity within the hydrogel network that enables a facile infiltration of MC3T3-E1 cells.^53^ Zhu et al.^88^ incorporated MG-63 and ST2 cells into ADA-GEL-based bioinks in order to investigate the cell activities after 3D bioprinting. Their results showed optical densities between 0.4 and 0.7 for MG-63 and 0.15 – 0.2 for ST2 cells after 7 days of incubation, which are comparable to the values measured for MC3T3-E1 cells in our 3D bioprinting study. Zehnder et al.^89^ evaluated the cellular activity of MG-63 cells within ADA-GEL scaffolds over 28 days of incubation. They measured an optical density of around 0.2 after 14 days of incubation.^89^ In our study, comparable activities were reached already after 7 days of incubation indicating the beneficial effect of the addition of FA^91^ and MBGNs.^33^ Monavari et al.^33^ seeded MC3T3-E1 cells on top of 3D printed ADA-GEL-based constructs composed of MBGNs and icariin. After a total incubation time of 6 days, the authors reported the highest viability after 24 h compared to 4 and 6 days of incubation. They explained their decrease in cell activity with the fast icariin release, that turned out to be cytotoxic for cells.^33^ However, in our study cells were directly incorporated into the inks and not seeded on top of 3D printed scaffolds, thus cell activities are difficult to compare. In our case the cell activity increases with increasing incubation time, which indicates the beneficial environment for cells provided by FA and MBGNs. Therefore, it can be concluded that the addition of FA and MBGNs is a promising approach to achieve a suitable environment for cell proliferation within 3D bioprinted ADA-GEL scaffolds. Besides that, the results confirm a suitable choice and adjustment of the printing parameters, which ensure a good shape fidelity of 3D printed scaffolds without harming the cells during the printing process.

### Vascular endothelial growth factor-A (VEGF-A) release

**Figure 10.**
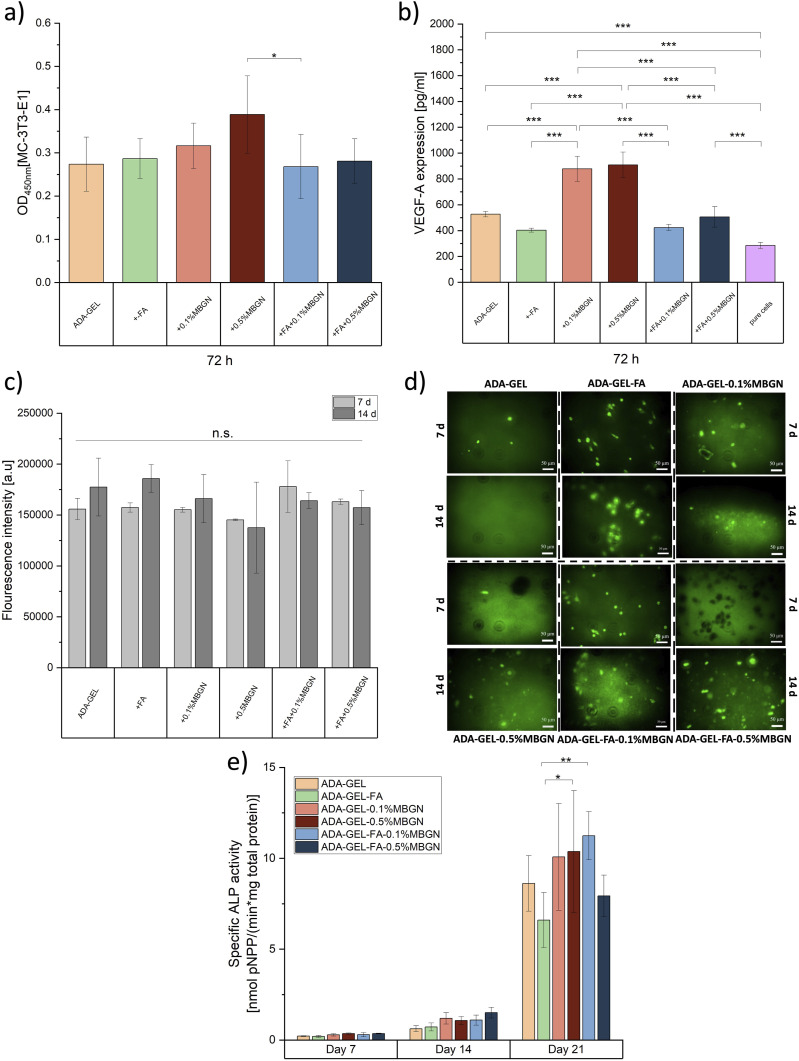
(a) Cell activity of the MC3T3-E1 cells seeded on top of all hydrogel compositions after 72 h of incubation. (b) VEGF-A expression of seeded MC3T3-E1 cells on top of all ADA-GEL composition covered with cell culture medium after 72 h of incubation. Pure cells seeded on cell culture plastic were used as a control group. (c) MC3T3-E1 cell-induced biomineralization seeded on all hydrogels after 7 and 14 days of incubation represented by fluorescence intensity. (d) Fluorescence images of green-coloured phases illustrating the MC3T3-E1 cell-induced biomineralization of the surface of all samples. Scale bar: 50 µm. (e) ALP activity of the MC3T3-E1 cells undergoing differentiation on top of all bioinks after 7, 14 and 21 days of incubation. Statistical analysis was performed via one-way ANOVA using the Bonferroni test with *p* < 0.05 = *, *p* < 0.01 = ** and *p* < 0.001 = ***.

The VEGF-A is a crucial biomolecule in the angiogenesis process^88^ and is secreted from certain cells including bone marrow stromal cells, fibroblasts and osteoblasts.^99^ In particular, VEGF-A is produced by cells that stimulate the formation of new blood vessels.^88^ As reported by Zhu et al.^88^ the relationship between vascularization and new bone formation is very close in bone TE. The induction of vascularization is an important signal for successful bone regeneration.^100^ Therefore, investigating whether FA or MBGNs within ADA-GEL-based hydrogels can enhance angiogenesis and provide a better environment for bone regeneration is of highest importance. The VEGF-A release by MC3T3-E1 cells was measured in this work. Briefly, 15,000 MC3T3-E1 cells were seeded on top of each ADA-GEL-based composition (listed in Table 1, *N* = 3) into each well of a 24-well plate for 72 h. After the incubation time the cell culture medium was collected to investigate the VEGA-A expression using a mouse VEGF-A Elisa kit. To ensure the activity of MC3T3-E1 cells on the day when samples were collected a following WST-8 assay was performed. In Figure 10(a) the WST-8 results (optical densities) are illustrated confirming a high cell activity after 72 h of incubation. In Figure 10(b) the impact of FA and MBGNs on the MC3T3-E1 VEGF-A secretion in pg/ml is shown. Pure cells, which were seeded on top of a blank well plate without any hydrogel were used as control group and exhibited a VEGF-A expression of 290 ± 24 pg/mL. The VEGF-A secretion induced by ADA-GEL, ADA-GEL-FA-0.1%MBGN and ADA-GEL-FA-0.5%MBGN samples are in a comparable high range of 528 ± 21 pg/mL, 424 ± 24 pg/mL and 506 ± 80 pg/mL, respectively. Interestingly, the VEGF-A secretion induced by pure ADA-GEL-FA samples showed the lowest VEGF-A secretion with 403 ± 15 compared to all other compositions. The secretion induced by ADA-GEL-0.1%MBGN and ADA-GEL-0.5%MBGN samples reached the highest values with 878 ± 98 pg/mL and 909 ± 99 pg/mL, respectively. The slightly lower VEGF-A expression of FA containing samples might be explained by the fact that FA acts as a tissue cell protector, inhibiting crucial inflammatory factors, such as VEGF.^95,101^ However, there are also studies, e.g. by Lin et al.^102^ and Li et al.,^103^ which reported an increase in VEGF expression owing to the presence of the phenolic compound FA. Briefly, Lin et al.^102^ investigated the effect of FA on angiogenesis through the modulation of endothelial cells and showed an enhanced VEGF expression.^102^ Li et al.^103^ observed an increased expression of VEGF in a rat model following pretreatment of neural stem cells with FA.^103^ Wang et al.^104^ noted an enhancement in angiogenesis by incorporating FA into electrospun matrices containing endothelial cells, when being combined with Astragaloside IV (AT) in a ratio of AT:FA of 7:3.^104^ AT was reported to increase the VEGF expression.^105^ In our study, the slight decrease of VEGF-A expression induced by FA containing samples is not significantly different, and the concentration is still higher than for pure cells indicating no significant negative effect of FA on VEGF-A expression. Additionally, since the concentration of FA is proportionally low compared to the MBGN content, the impact of MBGNs is likely higher on VEGF-A secretion than that of FA. Several studies claim that the upregulation of the angiogenic growth factor is due to the silicon ions present in MBGNs.^77,106^ Moreover, the release of Ca ions from MBGNs may improve the angiogenic effect, as reported by Kong et al.^107^ Overall, it should be highlighted that in all tested hydrogel compositions the VEGF-A expression was higher compared to that of pure cells (control group). Therefore, it can be concluded that the addition of FA has no negative impact while MBGNs significantly increased the VEGF-A expression. Since FA can act as an anti-angiogenic factor, the presence of MBGNs can counteract its action.^108^ Hence, a simultaneous incorporation of FA and MBGNs seems to be promising for bone TE, as VEGF-A is considered to be the most important molecule regulating vascular development.^109^

### Alkaline phosphatase (ALP) activity and Bradford analysis

ALP is a marker for the activity of osteoblasts and its expression can prove osteogenic differentiation.^81,92^ To assess the impact of FA and MBGNs on the osteogenic differentiation of pre-osteoblastic MC3T3-E1, an ALP/Bradford analysis was carried out. The specific ALP activity of seeded MC3T3-E1 cells on the surface of each hydrogel (compositions mentioned in Table 1) was quantified to evaluate the osteogenic differentiation ability after 7, 14 and 21 days of incubation. As can be seen in Figure 10(e), compared to day 7, all scaffolds provoke a significant increase of ALP activity confirming an osteogenesis ability on day 21. Among all compositions, ADA-GEL-FA appeared to lead to the lowest differentiation after 21 days (6.60 ± 1.52 nmol pNpp/min*mg), which might be due to the higher resistance to degradation and thus reduded GEL release of the FA containing hydrogel compared to neat ADA-GEL.^33,48^ After 7 days of incubation, ADA-GEL-FA-0.5%MBGN shows the highest differentiation with 1.51 ± 0.29 nmol pNpp/min*mg in comparison to other compositions. However, this behavior changed after 21 days, which might be explained by the high release of Ca ions in ADA-GEL-FA-0.5%MBGN after 14 days (see section Ca release), leading to an alkalinization of the medium. The alkalinization might negatively affect cells resulting in less differentiation on day 21 compared to other compositions. Similar results were reported by Monavari et al.^33^ for ADA-GEL samples with MBGNs, which also released a sufficient concentration of Ca ions leading to an alkalinized medium and less differentiation compared to other tested materials. Briefly, in their study they also worked with ADA-GEL and MBGNs and showed a relatively low ALP activity for day 7 (<0.01 nmol pNpp/min*mg) with an increase to a maximum of 0.8 nmol pNpp/min*mg on day 14, values which are comparable with the values for our samples up to 14 days. Our results for day 21 showed for all compositions an ALP activity between 8 and 11 nmol pNpp/min*mg. Interestingly, the ADA-GEL-FA-0.1%MBGN composite seems to express the highest osteogenic differentiation, which reached a value of 11.24 ± 1.32 nmol pNpp/min*mg. This result is confirmed by Hsu et al.,^110^ who incorporated MBG nanofibers into polymeric scaffolds and incubated them with MG-63 osteoblast-like cells. Their results revealed that the addition of the MBGNs significantly increased the ALP activity in comparison to the reference material. They claimed that this behaviour might be related to the Ca ions released by the nanoparticles.^110^ Other studies confirmed that MBGNs act as carriers of biologically required ions, such as Ca and Si, to create an environment that stimulates osteogenic differentiation, which is of the highest interest for bone repair.^88^ Moreover, Jung et al.^111^ conducted a study specifically with MC3T3-E1 cells proving that Ca ions can stimulate their differentiation due to the activation of specific L-Type Ca channels.^111^ Comparing the results, which were established from the Ca release study on day 21, the concentration of Ca ions released was the highest for ADA-GEL-FA-0.1%MBGN and ADA-GEL-FA-0.5%MBGN, which can explain the higher ALP activity compared to neat ADA-GEL and ADA-GEL-FA samples. Moreover, the release of Si ions from MBGNs can also lead to an increase of ALP activity due to an induction of osteoblast differentiation, shown for example by Reffitt et al.^112^ and Keeting et al.^113^ Interestingly, in our study, FA does not seem to have a high impact on osteoblast differentiation, even though FA was found to increase the expression of ALP and other osteogenic markers (Runx-2, OSX, Col-I, OSN) on MC3T3-E1 cells^114^ and bone marrow-derived MSCs.^115^ This might be due to the relatively low concentration of FA compared to MBGNs in our samples resulting in a higher impact of MBGNs on the ALP activity.

### *In vitro* biomineralization

The quantification of mineral content was performed utilizing the OsteoImage Mineralization Assay (Lonza, Germany), a method designed to selectively bind with HAp nodules. This in vitro assay enables the precise measurement of bone cell mineralization and is based on the specific interaction between the fluorescent OsteoImage staining reagent and the HAp components within the bone-like nodules produced by cells.^116^ The accumulation of HAp deposits originating from pre-osteoblasts MC3T3-E1, which were stimulated by a mineralization medium containing β-glycerophosphate and ascorbic acid, was observed and quantified. This detection and quantification were accomplished through fluorescent staining of samples after 7 and 14 days of incubation, as shown in Figure 10(d). After 14 days of cell culture, the FA containing ADA-GEL-MBGN samples appeared to show more green-coloured mineralization phases compared to hydrogels without FA or neat ADA-GEL samples. This is also confirmed by the quantitative analysis presented in Figure 10(c) which shows the highest fluorescence intensity for ADA-GEL-FA-0.1%MBGN. These high values of absorbance suggest a high in vitro mineralization over time. In some cases, black phases are visible which might be explained by the aggregation and sedimentation of some undistributed MBGNs within the hydrogel and might affect the detection of green-coloured phases. In general, ions released from MBGNs into body fluids stimulate the creation of hydroxycarbonate apatite (HCA) and activate genes responsible for promoting osteogenesis.^48,62^ It seems that this property might be enhanced due to the incorporation of FA to ADA-GEL-MBGN samples. The positive impact of the addition of a phytotherapeutic agent to ADA-GEL hydrogels was also reported by Monavari et al.,^33^ who loaded MBGNs with icariin and showed a higher mineralization level compared to pure ADA-GEL samples. However, these results were obtained by staining 3D printed constructs instead of compact films as it was done in our work. Therefore, the detection of green-coloured phases is difficult to compare.^33^ Ghorbani et al.^117^ showed comparable green-coloured phases for ADA-GEL-based and ADA-GEL samples coated with polydopamine by using the same mineralization assay, confirming the presence of mineral phases in ADA-GEL-based samples.^117^ In Figure 10(d) one can observe that for neat ADA-GEL samples fewer green-coloured phases were detected indicating the positive effect of MBGNs and FA on the in vitro biomineralization of ADA-GEL-FA-MBGN samples.

## Conclusions

This work successfully introduced FA and MBGNs as effective additives for ADA-GEL-based hydrogels, designed for applications in bone TE and 3D (bio)printing. The degradation and swelling characteristics of both films and 3D printed scaffolds showed an accelerated trend with an increase in MBGN content. Conversely, the presence of FA appeared to impart stability to the samples. Release studies of FA, GEL, and Ca ions further validated the delivery capabilities of ADA-GEL samples. The increased viscosity of ADA-GEL-FA and ADA-GEL-FA-0.1%MBGN inks explained the favourable behaviour of these inks for 3D printing, manifested by the suitable shape fidelity of the printed scaffolds. Additionally, the internal crosslinking between FA and GEL, leading to a higher degree of crosslinking, enhances the printability properties compared to pure ADA-GEL hydrogel without any additives. This behaviour was additionally confirmed through various printing experiments. Moreover, for all samples, high cell viability and activity, especially with the most promising bioinks, were confirmed through direct cell tests and 3D bioprinting trials. Additionally, the incorporation of MBGNs improved the properties of ADA-GEL hydrogels in terms of their ability to create HAp on the surface of samples, enhancing mechanical properties and leading to the formation of mineral phases, as assessed by an in vitro biomineralization assay. MBGN addition also resulted in increased ALP activity and enhanced VEGF-A expression of MC3T3-E1 cells. Therefore, the incorporation of FA and MBGNs (at tailored concentrations) in ADA-GEL hydrogels demonstrates a convenient approach for applications in 3D (bio)printing within the field of bone TE. Clearly, the combination of different materials in a multimaterial composite hydrogel system results in a complex structure, which was extensively characterized in this work from a materials science perspective. Further in-depth characterization at the biomolecular level, particularly the local interaction between the ADA-GEL hydrogel molecules and FA and/or MBGNs, remains to be investigated in future studies.

## Supplemental Material


Supplemental Material - Enhancing alginate dialdehyde-gelatin (ADA-GEL) based hydrogels for biofabrication by addition of phytotherapeutics and mesoporous bioactive glass nanoparticles (MBGNs)
Supplemental Material for Enhancing alginate dialdehyde-gelatin (ADA-GEL) based hydrogels for biofabrication by addition of phytotherapeutics and mesoporous bioactive glass nanoparticles (MBGNs) by Faina Bider, Chiara Gunnella, Jana T Reh, Corina-Elena Clejanu, Sonja Kuth, Ana M Beltrán and Aldo R Boccaccini in Journal of Biomaterials Applications.

Supplemental Material - Enhancing alginate dialdehyde-gelatin (ADA-GEL) based hydrogels for biofabrication by addition of phytotherapeutics and mesoporous bioactive glass nanoparticles (MBGNs)
Supplemental Material for Enhancing alginate dialdehyde-gelatin (ADA-GEL) based hydrogels for biofabrication by addition of phytotherapeutics and mesoporous bioactive glass nanoparticles (MBGNs) by Faina Bider, Chiara Gunnella, Jana T Reh, Corina-Elena Clejanu, Sonja Kuth, Ana M Beltrán and Aldo R Boccaccini in Journal of Biomaterials Applications.
