# Supplementary material for: Enhancing alginate dialdehyde-gelatin (ADA-GEL) based hydrogels for biofabrication by addition of phytotherapeutics and mesoporous bioactive glass nanoparticles (MBGNs)
Source: J Biomater Appl. 2024 Sep 21;39(6):524–56. doi: 10.1177/08853282241280768 (PMC11707976; doi:10.1177/08853282241280768)
Supplement: Supplemental Material - Enhancing alginate dialdehyde-gelatin (ADA-GEL) based hydrogels for biofabrication by addition of phytotherapeutics and mesoporous bioactive glass nanoparticles (MBGNs) [file sj-pdf-1-jba-10.1177_08853282241280768.pdf]

6. Supplementary

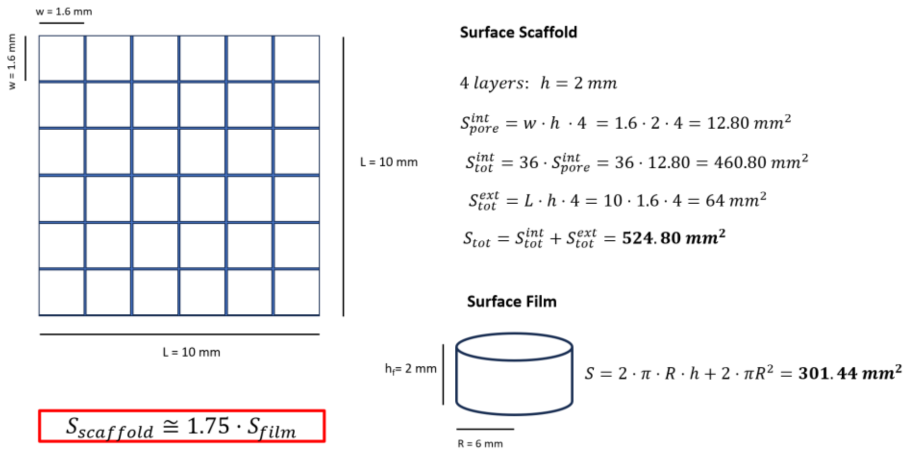

Figure S1: Rough estimation of the exposed surfaces of 3D printed scaffolds compared to films.

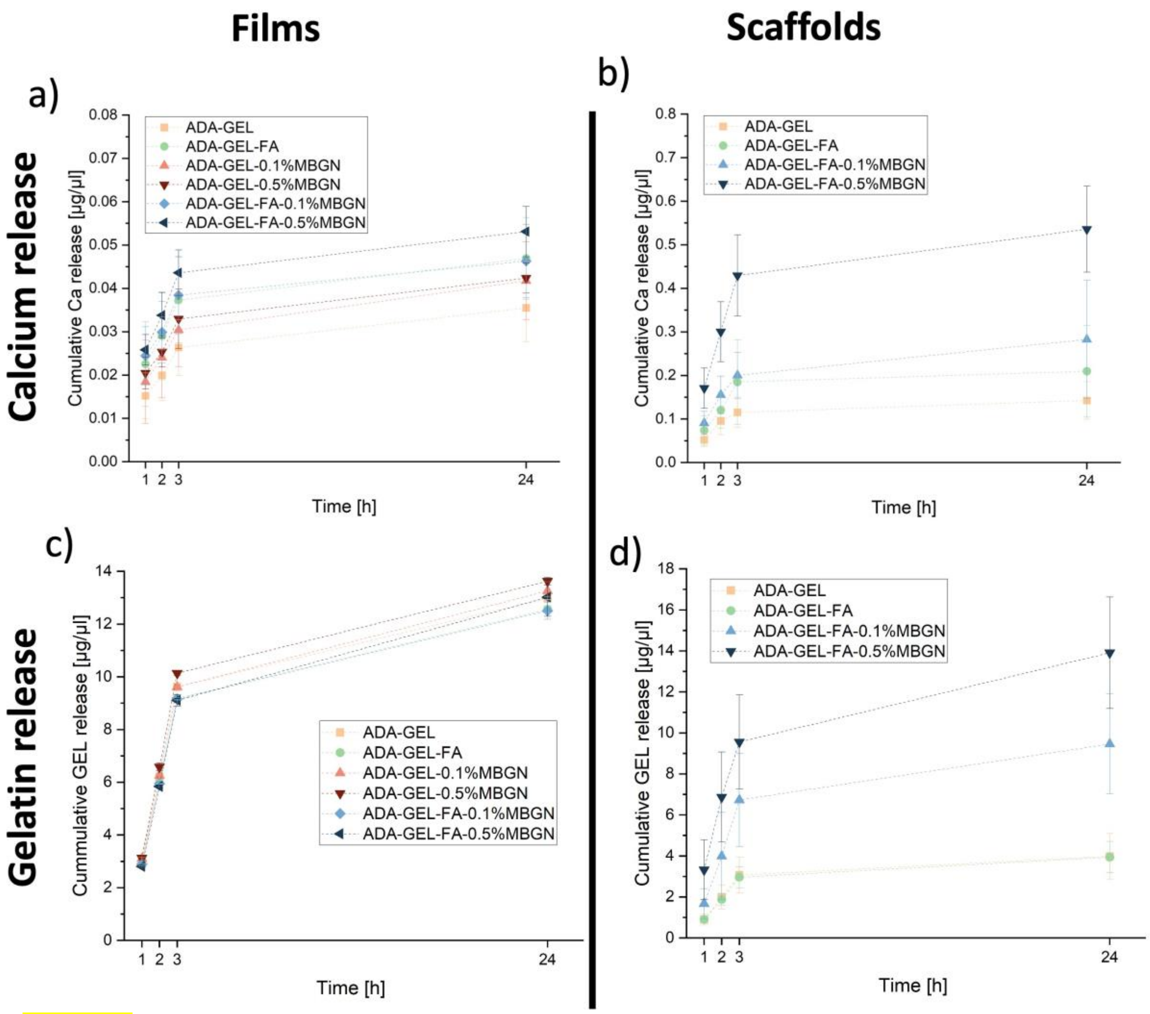

Figure S2: First hours of cumulative Ca release out of a) films of all used hydrogel compositions and b) 3D printed ADA-GEL scaffolds with/without FA and MBGNs in [µg/µl]. First hours of cumulative GEL release out of c) films of all used hydrogel compositions and d) 3D printed ADA-GEL scaffolds with/without FA and MBGNs in [µg/µl].

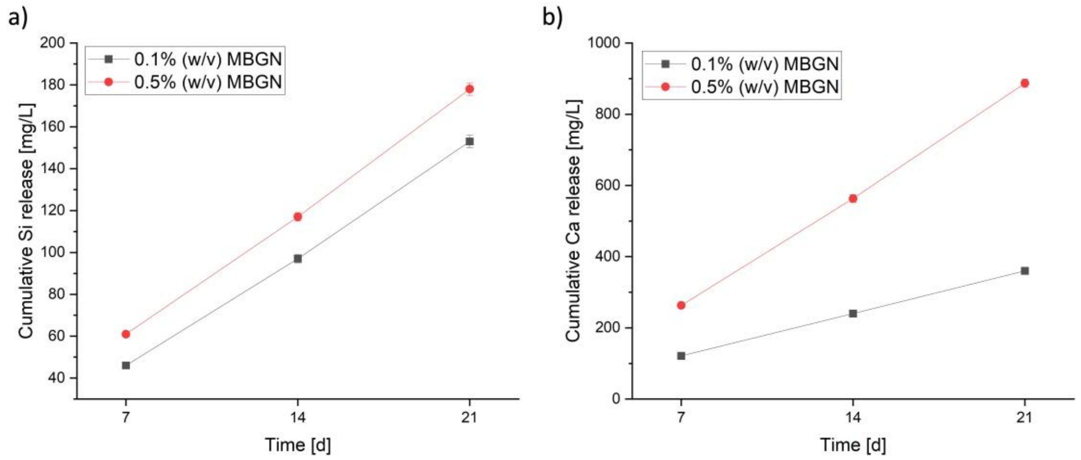

Figure S3: Cumulative and Si and Ca-ions release of MBGNs after 7, 14 and 21 days of incubation in SBF solution determined by optical emission spectroscopy with inductively coupled plasma (ICP-OES).

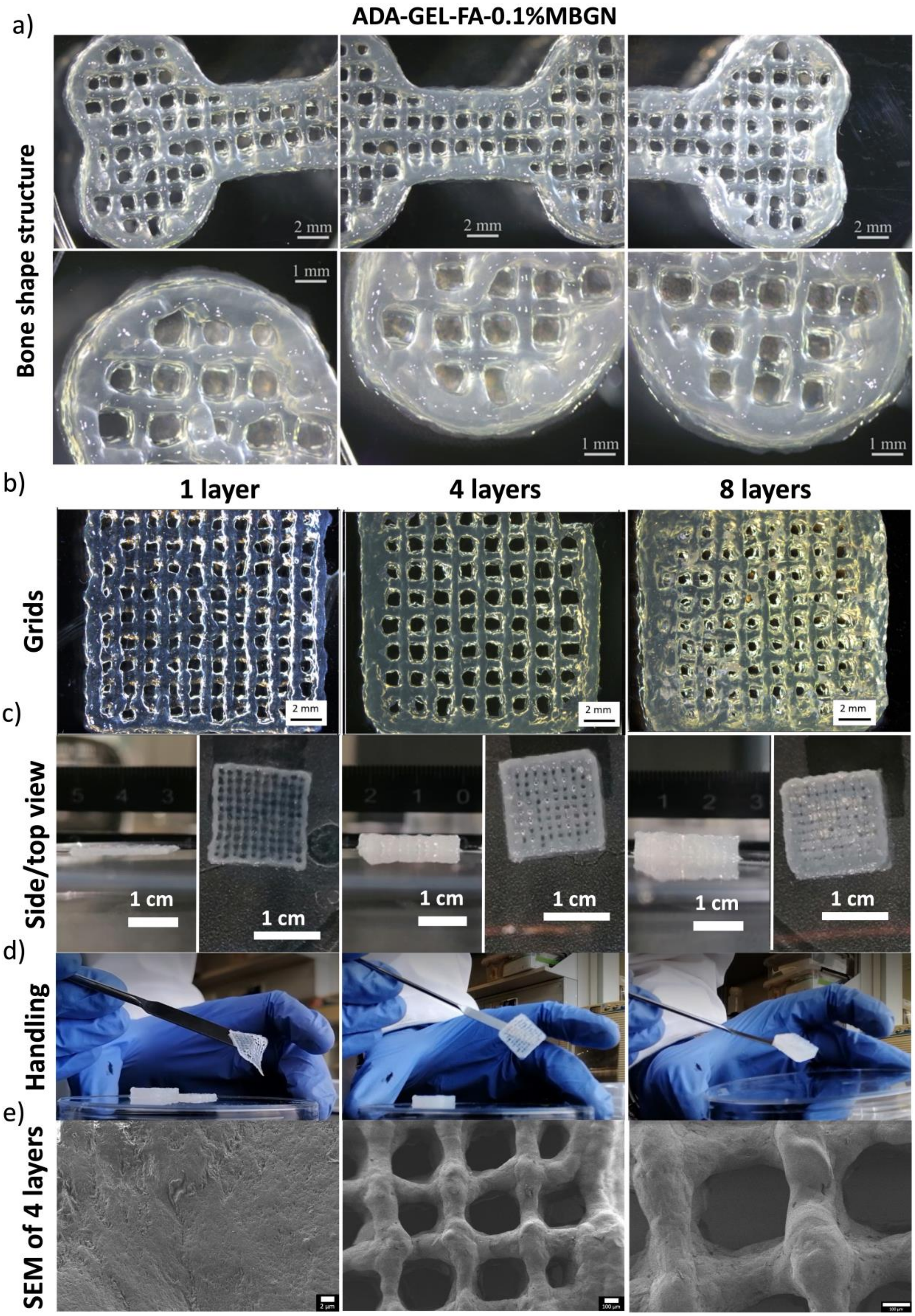

Figure S4: More complex printing with most promising ADA-GEL-FA-0.1%MBGN ink. a) Microscopy images of bone shape structure. Scale bars: 1 mm and 2 mm. b) Microscopy images of 1.5 x 1.5 cm^2 grids printed with 1, 4 and 8 layers. Scale bar: 2 mm. c) Photos of top view and side view of grids. Scale bar: 1 cm. d) Photos confirming the good handling of 3D printed scaffolds. e) SEM images of 4 layers printed scaffold. Scale bar: 2 µm and 100 µm.
