# Supplementary material for: Enhancing alginate dialdehyde-gelatin (ADA-GEL) based hydrogels for biofabrication by addition of phytotherapeutics and mesoporous bioactive glass nanoparticles (MBGNs)
Source: J Biomater Appl. 2024 Sep 21;39(6):524–56. doi: 10.1177/08853282241280768 (PMC11707976; doi:10.1177/08853282241280768)
Supplement: Supplemental Material - Enhancing alginate dialdehyde-gelatin (ADA-GEL) based hydrogels for biofabrication by addition of phytotherapeutics and mesoporous bioactive glass nanoparticles (MBGNs) [file sj-pdf-2-jba-10.1177_08853282241280768.pdf]

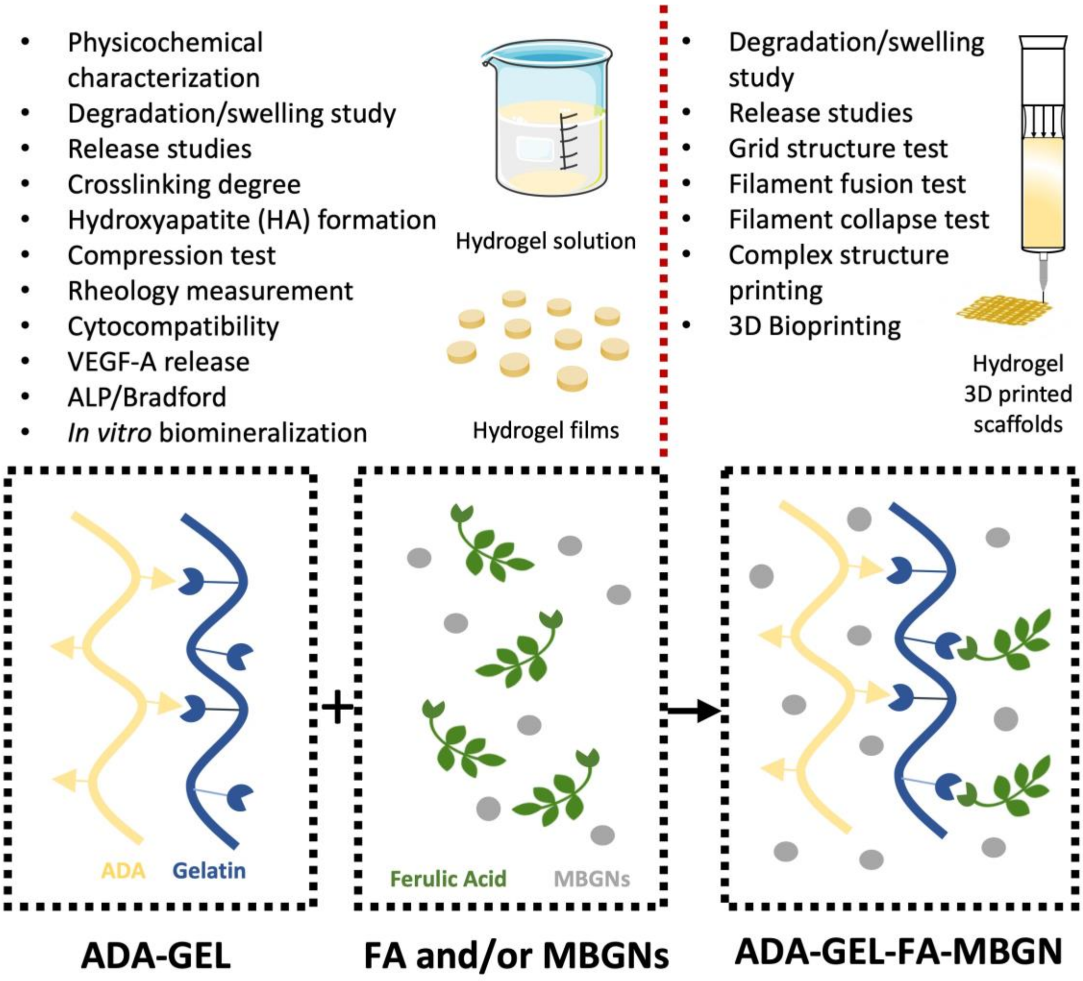

Physicochemical characterization
Degradation/swelling study
Release studies
Crosslinking degree
Hydroxyapatite (HA) formation
Compression test
Rheology measurement
Cytocompatibility
VEGF-A release
ALP/Bradford
In vitro biomineralization
Hydrogel solution
Hydrogel films
Degradation/swelling study
Release studies
Grid structure test
Filament fusion test
Filament collapse test
Complex structure printing
3D Bioprinting
Hydrogel 3D printed scaffolds
ADA
Gelatin
Ferulic Acid
MBGNs
ADA-GEL
FA and/or MBGNs
ADA-GEL-FA-MBGN
